# Supplementary material for: PKM2 Regulates HSP90-Mediated Stability of the IGF-1R Precursor Protein and Promotes Cancer Cell Survival during Hypoxia
Source: Cancers (Basel). 2021 Jul 30;13(15):3850. doi: 10.3390/cancers13153850 (PMC8345735; doi:10.3390/cancers13153850)
Supplement: Supplementary file 1 [file cancers-13-03850-s001.zip › PKM2-IGF1R-uncropped blots_2021-07-27.pdf]

Supplementary Materials

Fig. 1E

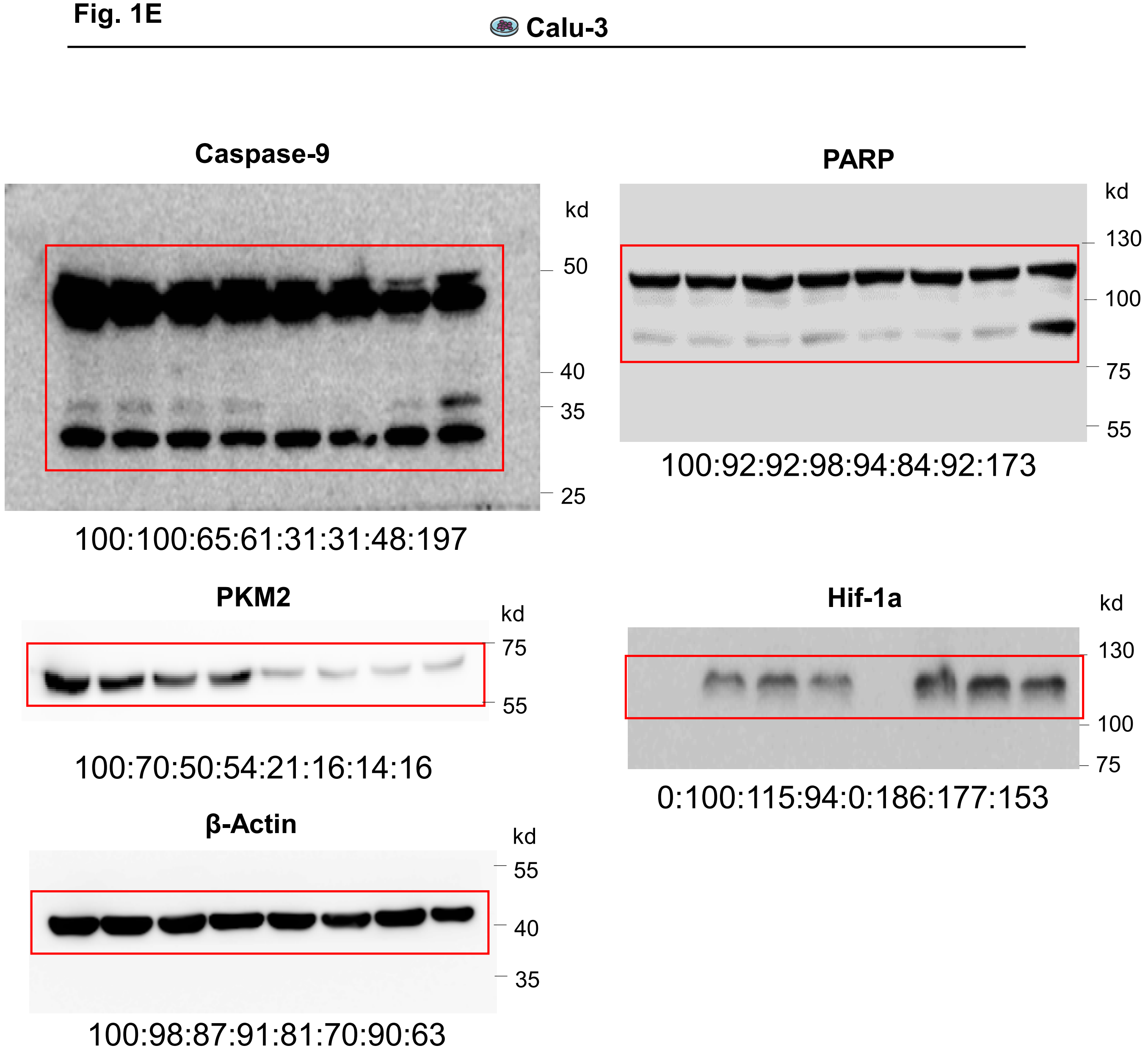

Full size immunoblots of cropped blots for Fig. 1E.

Fig. 2A

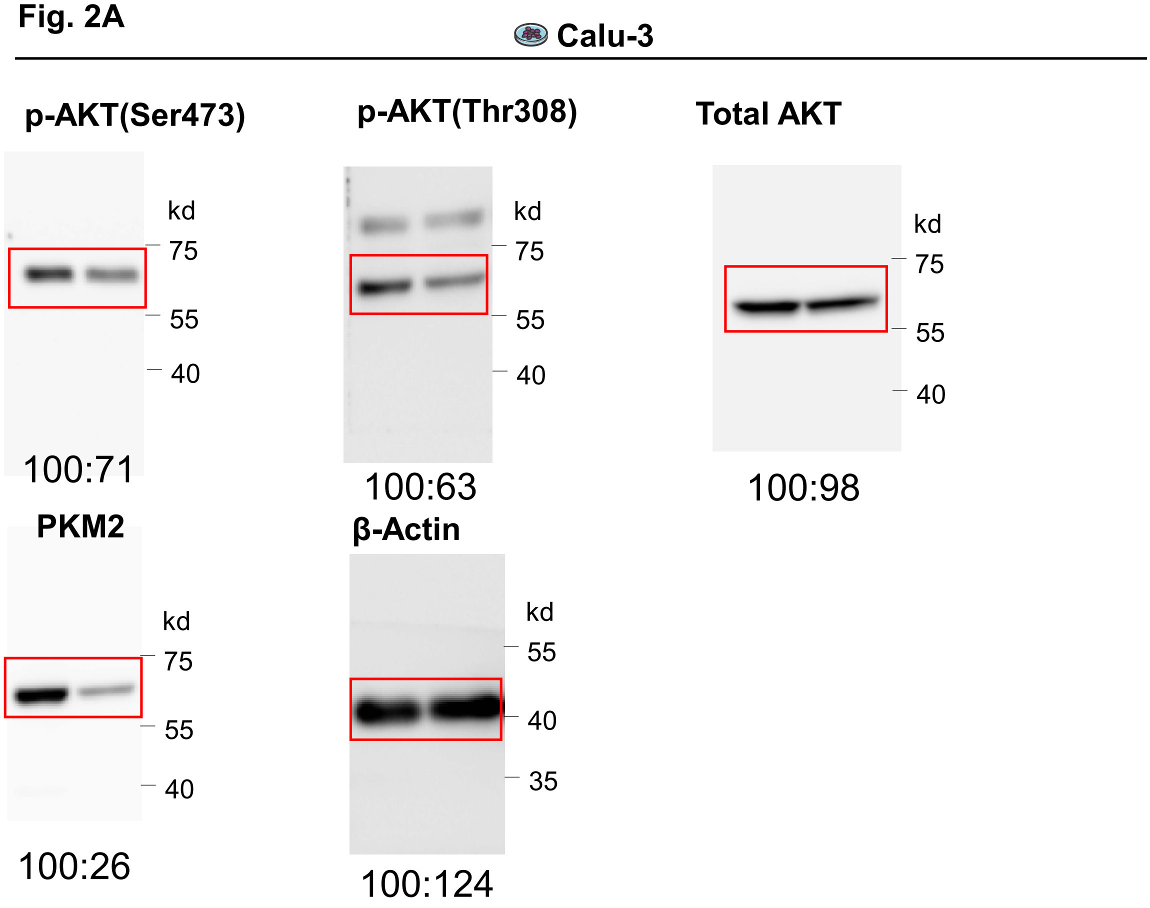

Fig. 2A

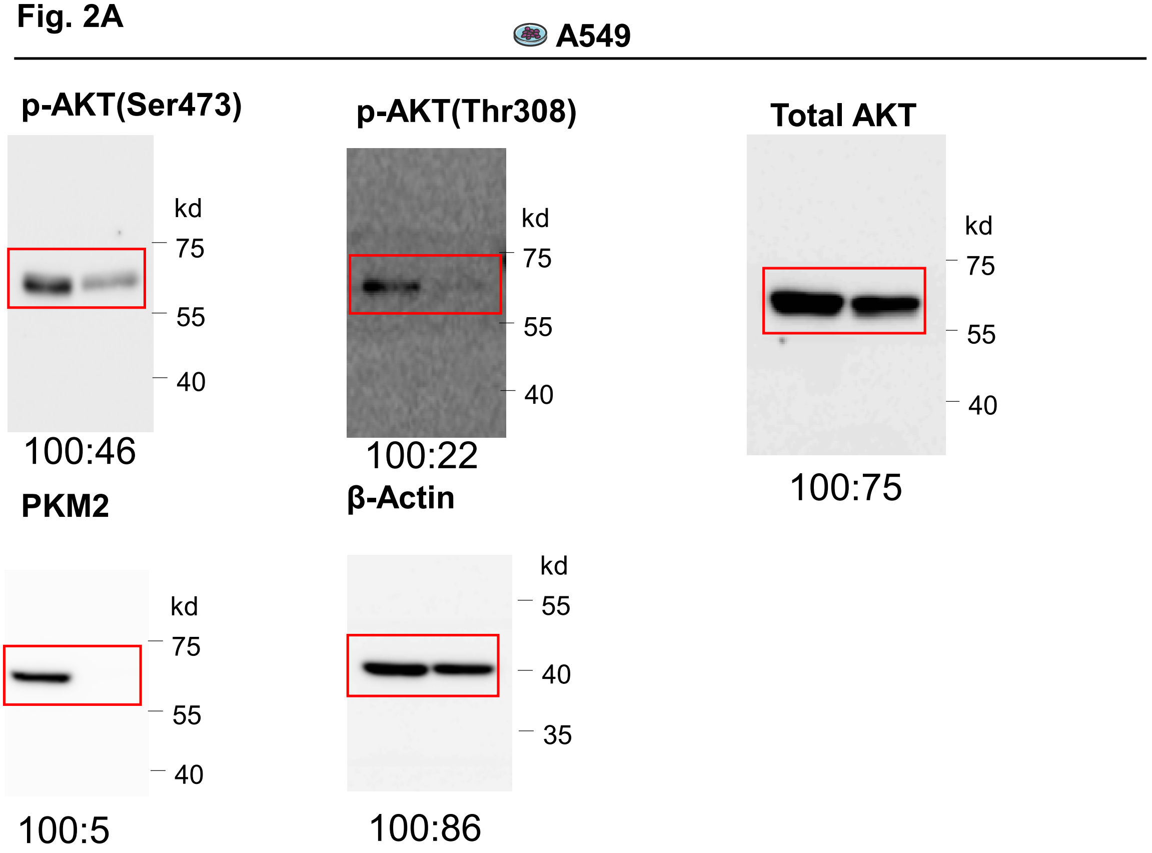

Full size immunoblots of cropped blots for Fig. 2A.

Fig. 2C

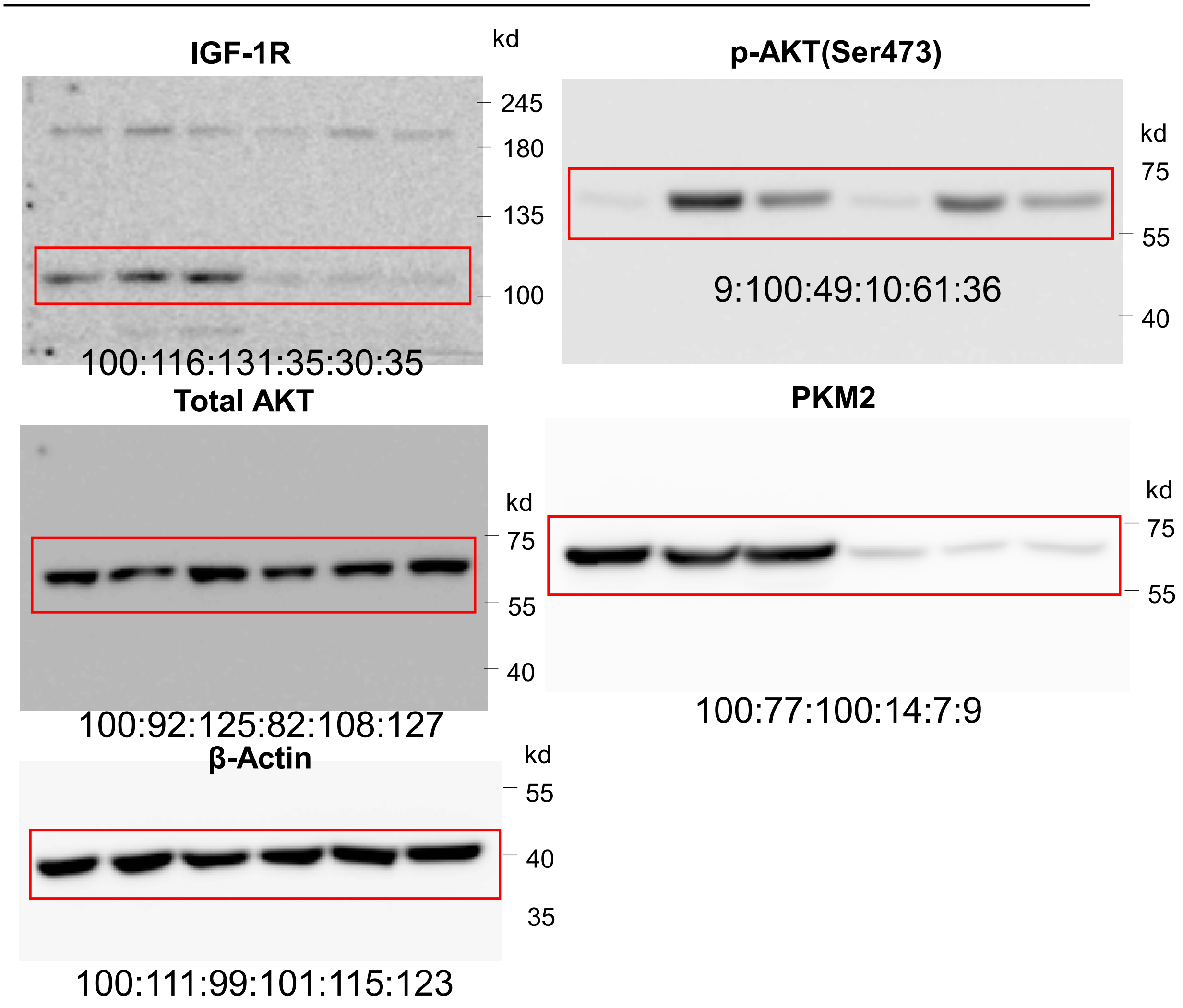

Fig. 2E

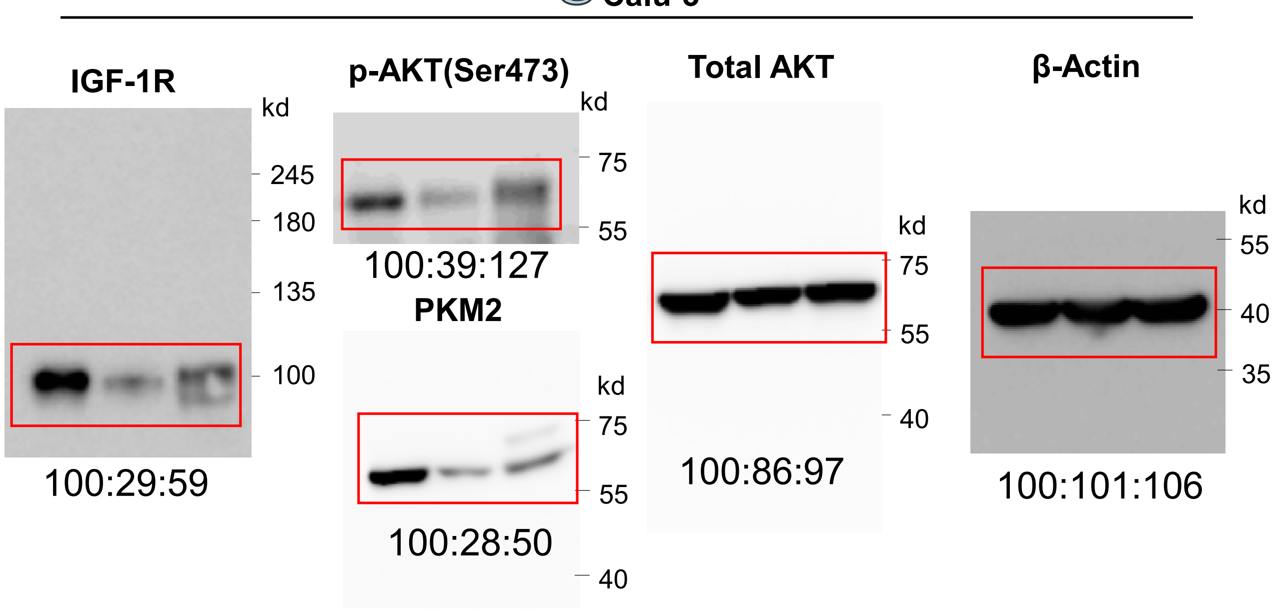

Full size immunoblots of cropped blots for Fig. 2C and 2E.

Fig. 2F

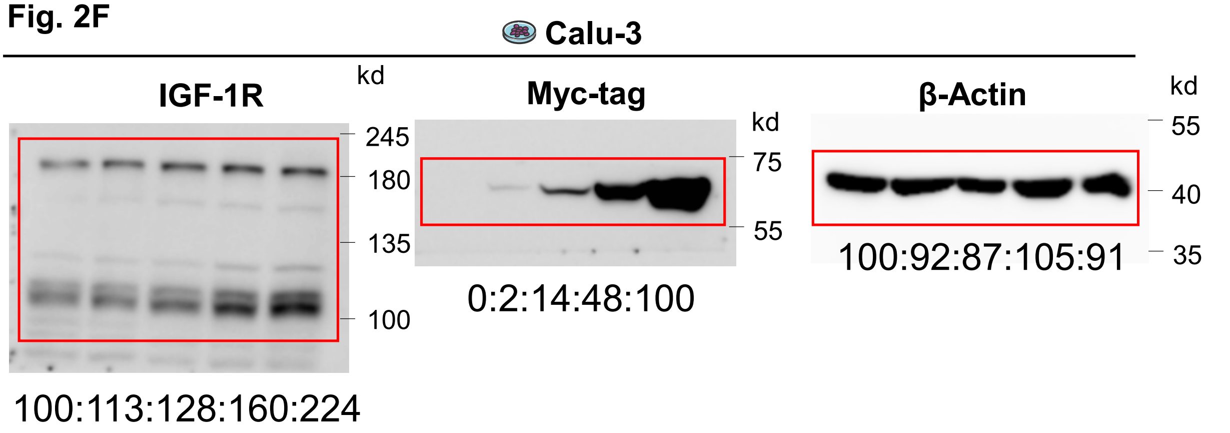

Fig. 2G

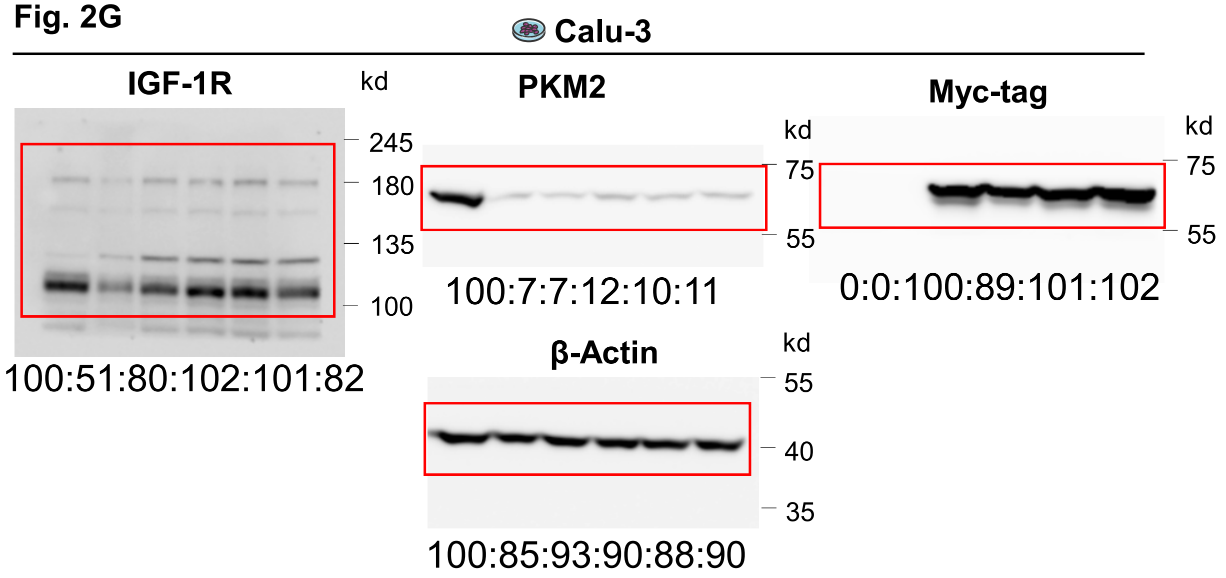

Fig. 3A

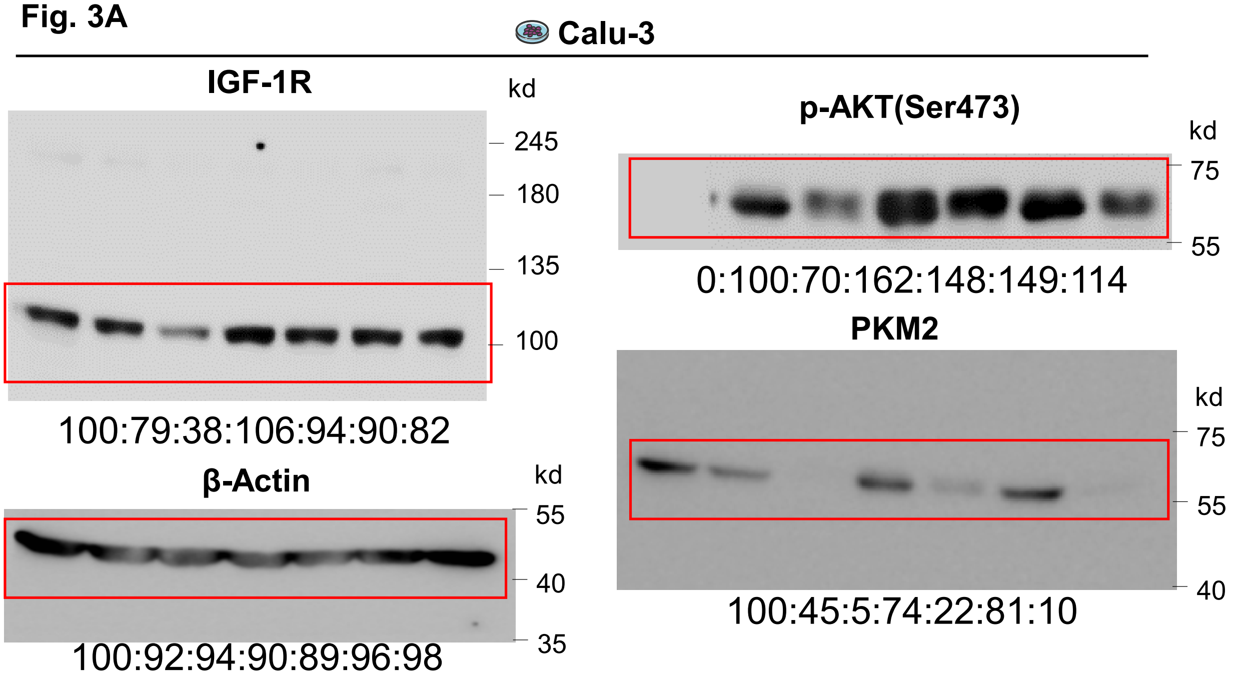

Full size immunoblots of cropped blots for Fig. 2F, 2G and 3A.

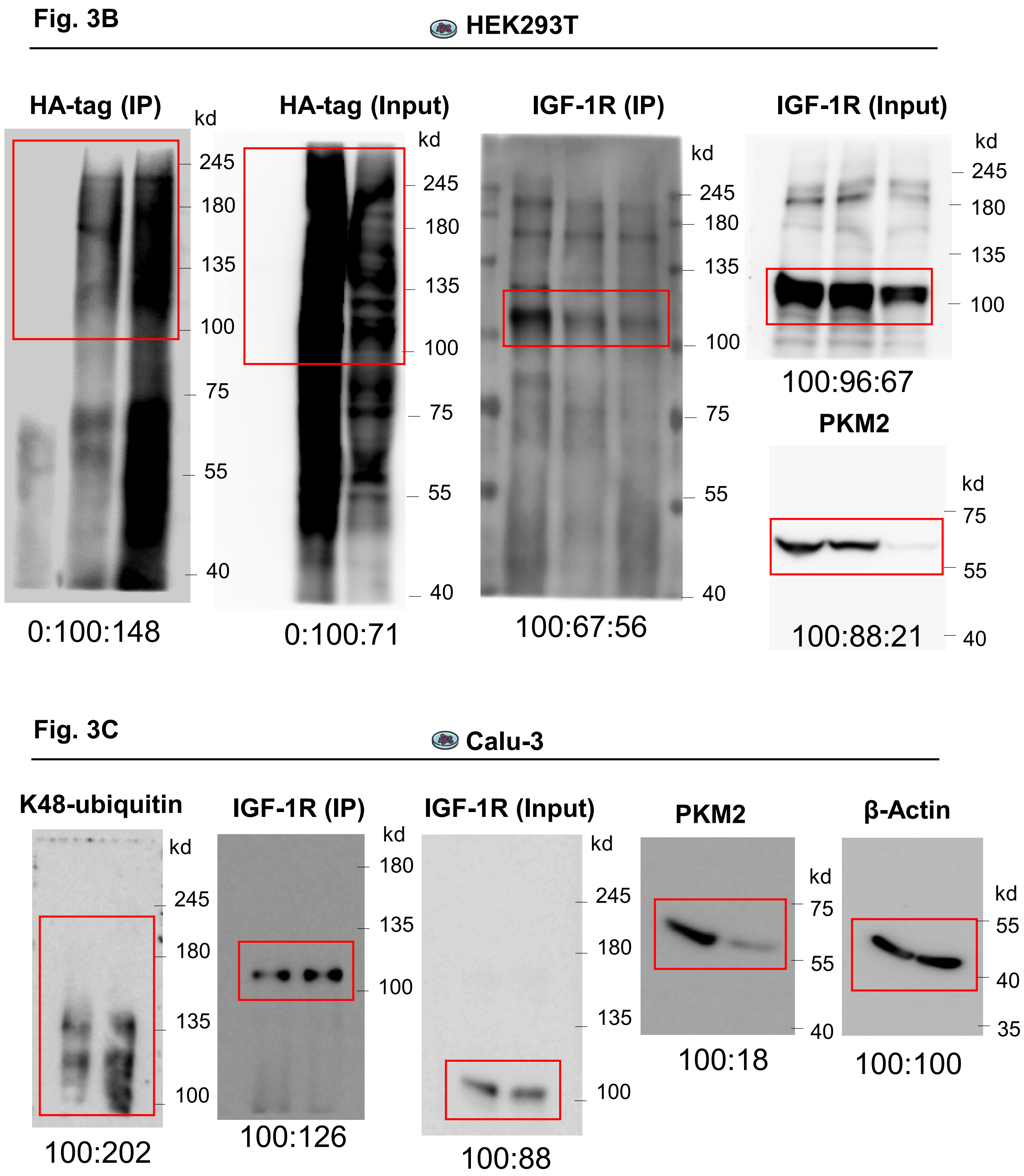

Full size immunoblots of cropped blots for Fig. 3B and 3C.

Fig. 3D

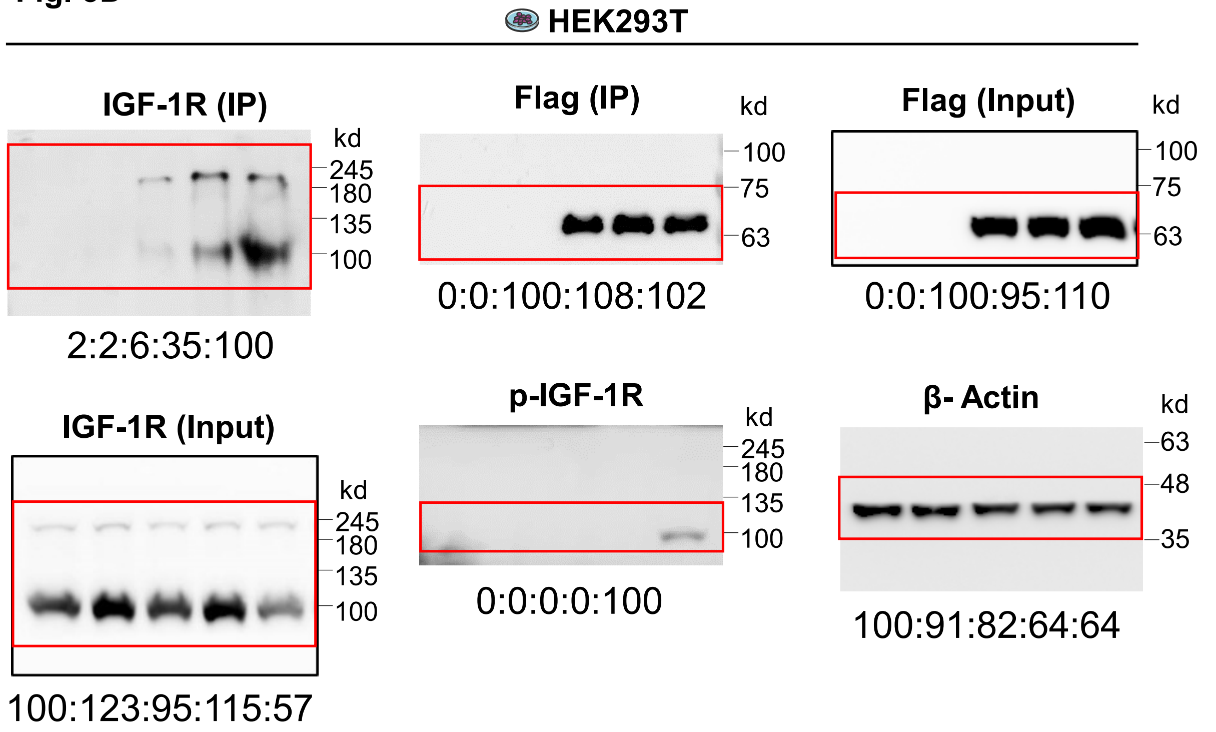

Fig. 3E

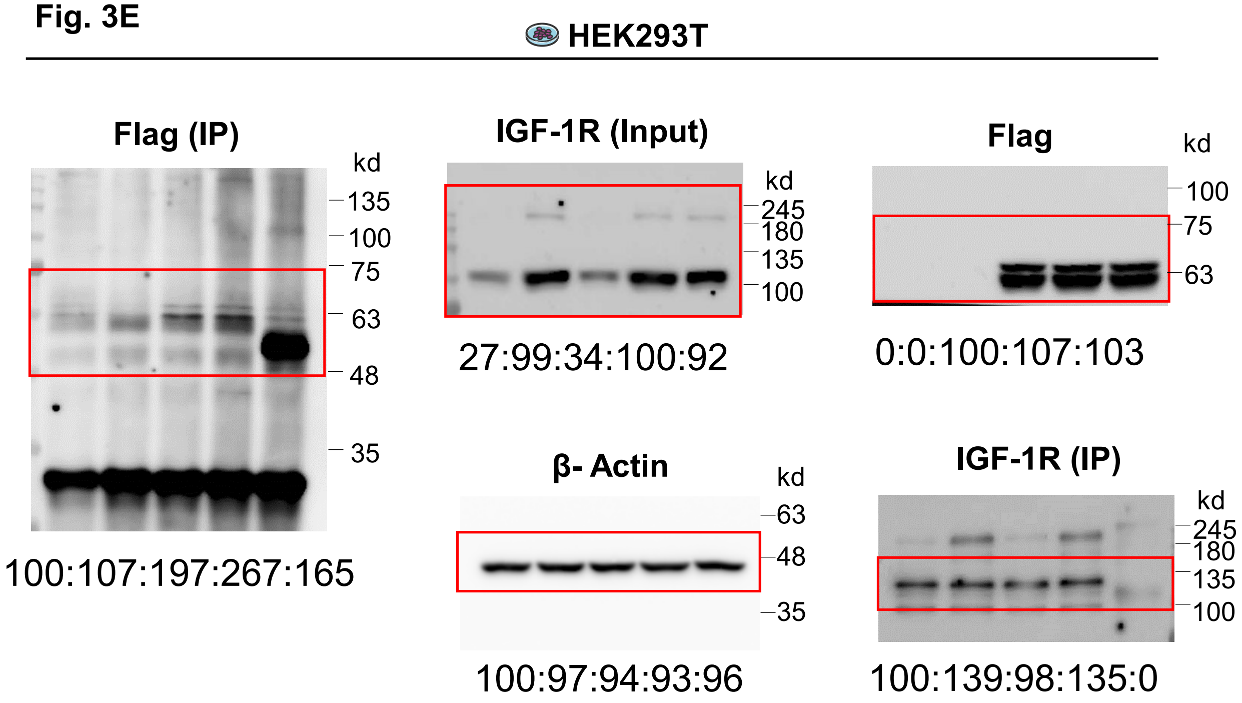

Full size immunoblots of cropped blots for Fig. 3D and 3E.

Fig. 3F

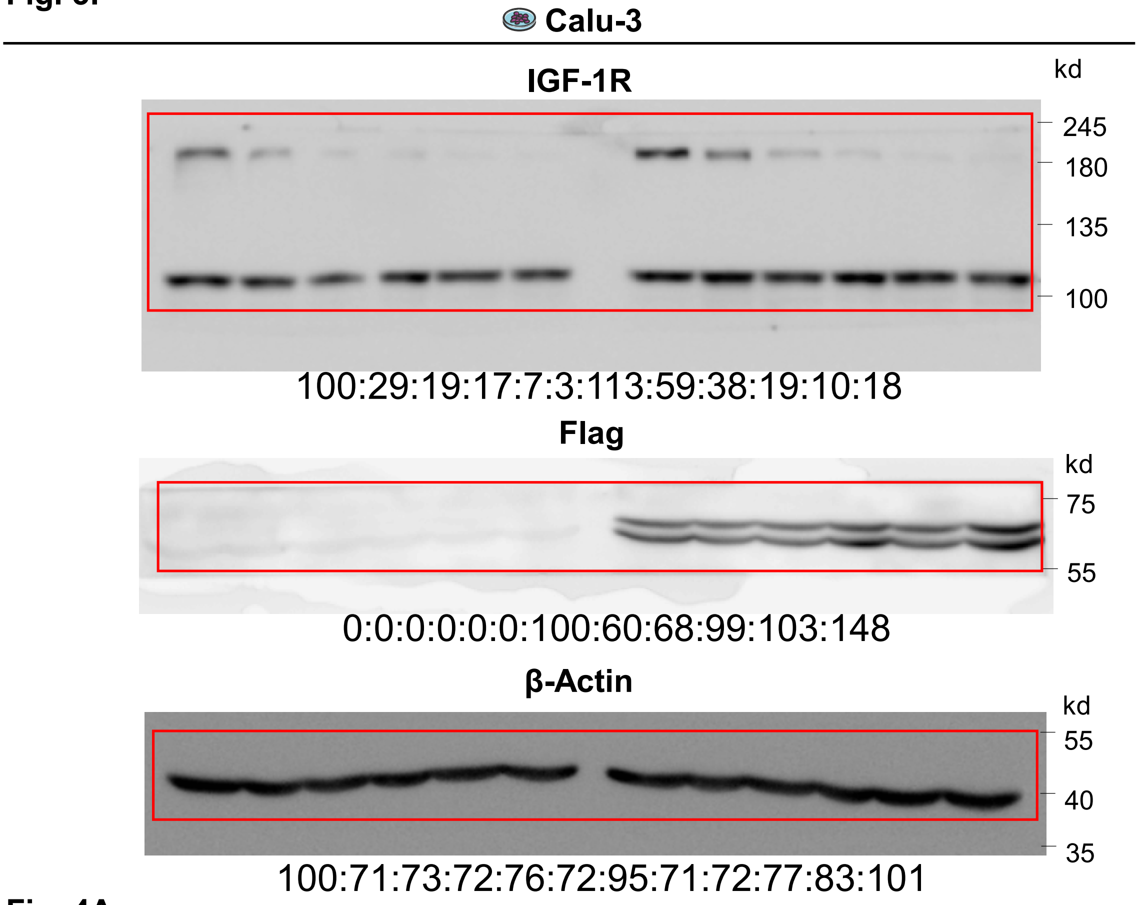

Fig. 4A

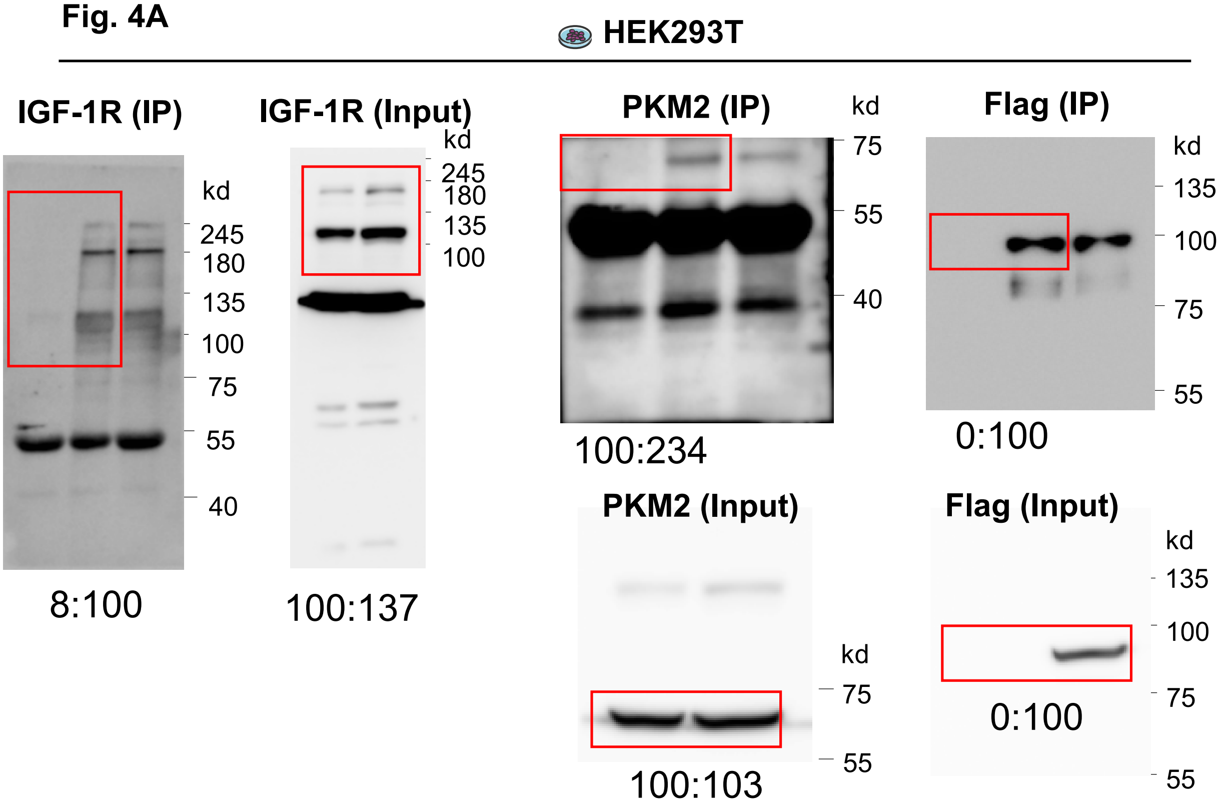

Full size immunoblots of cropped blots for Fig. 3F and 4A.

Fig. 4B

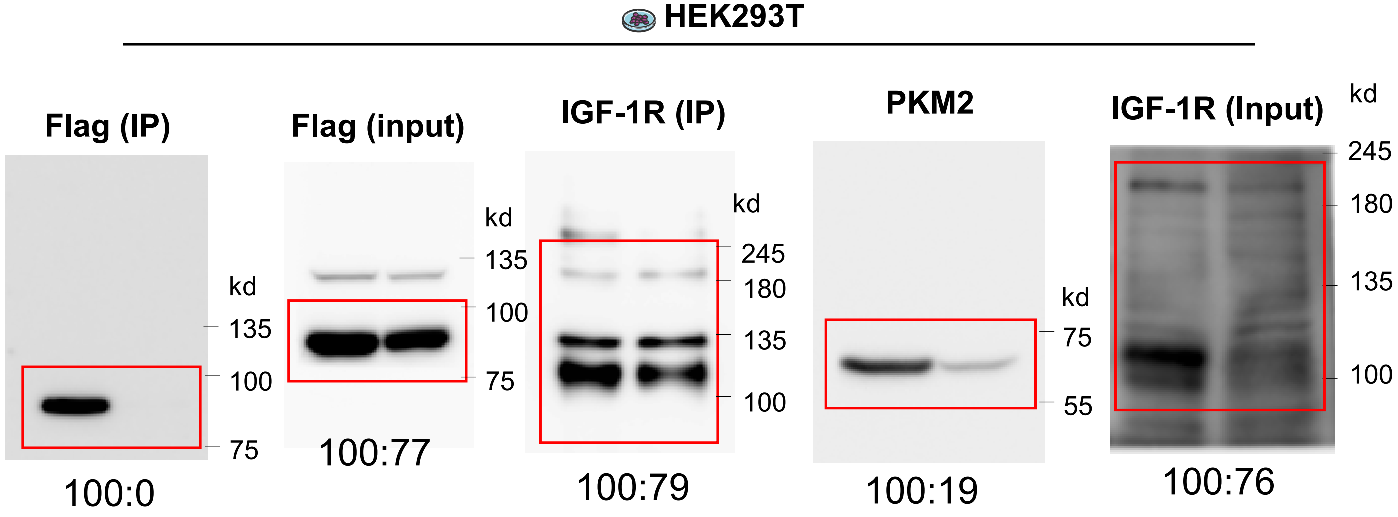

Fig. 4C

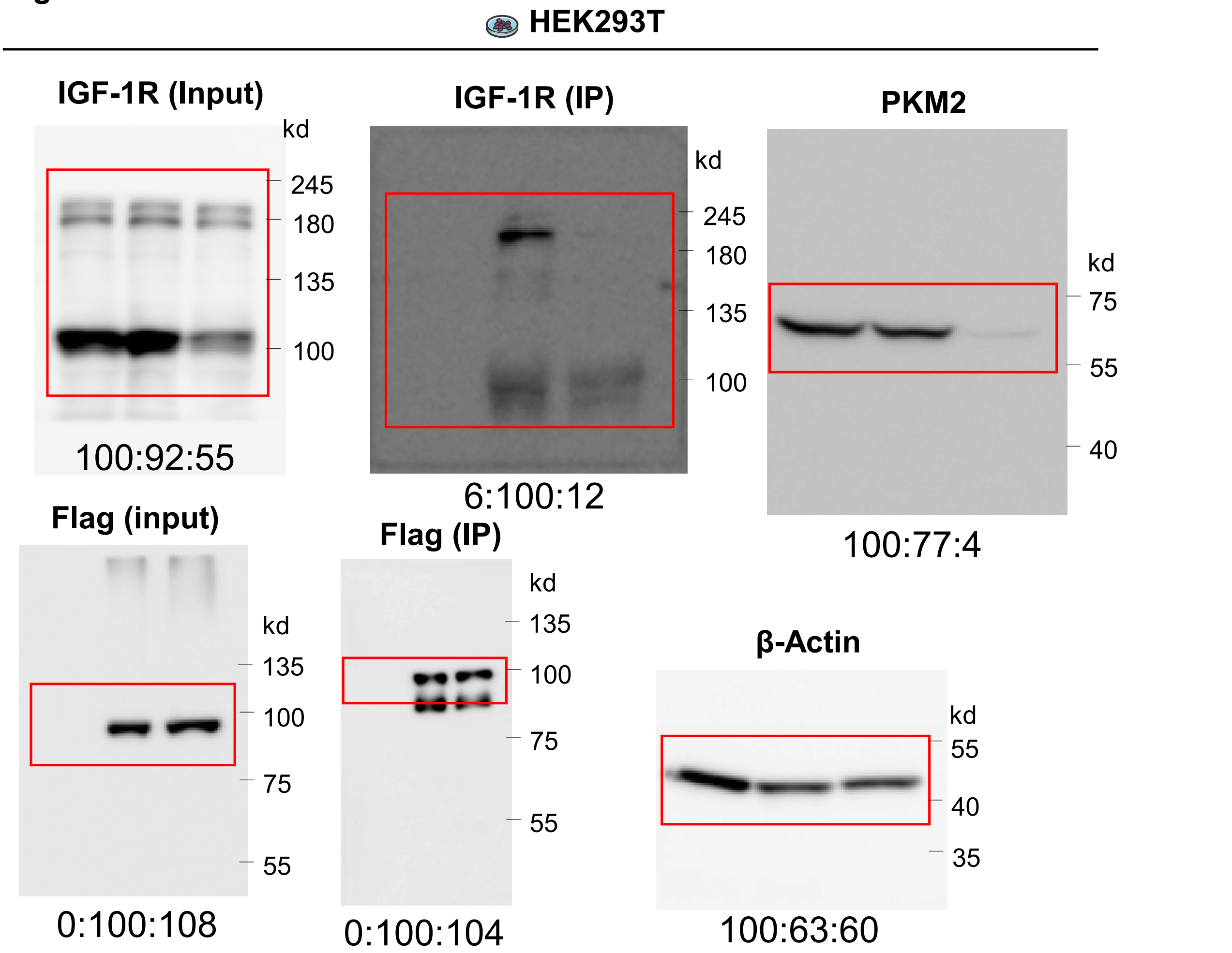

Full size immunoblots of cropped blots for Fig. 4B and 4C.

Fig. 4D

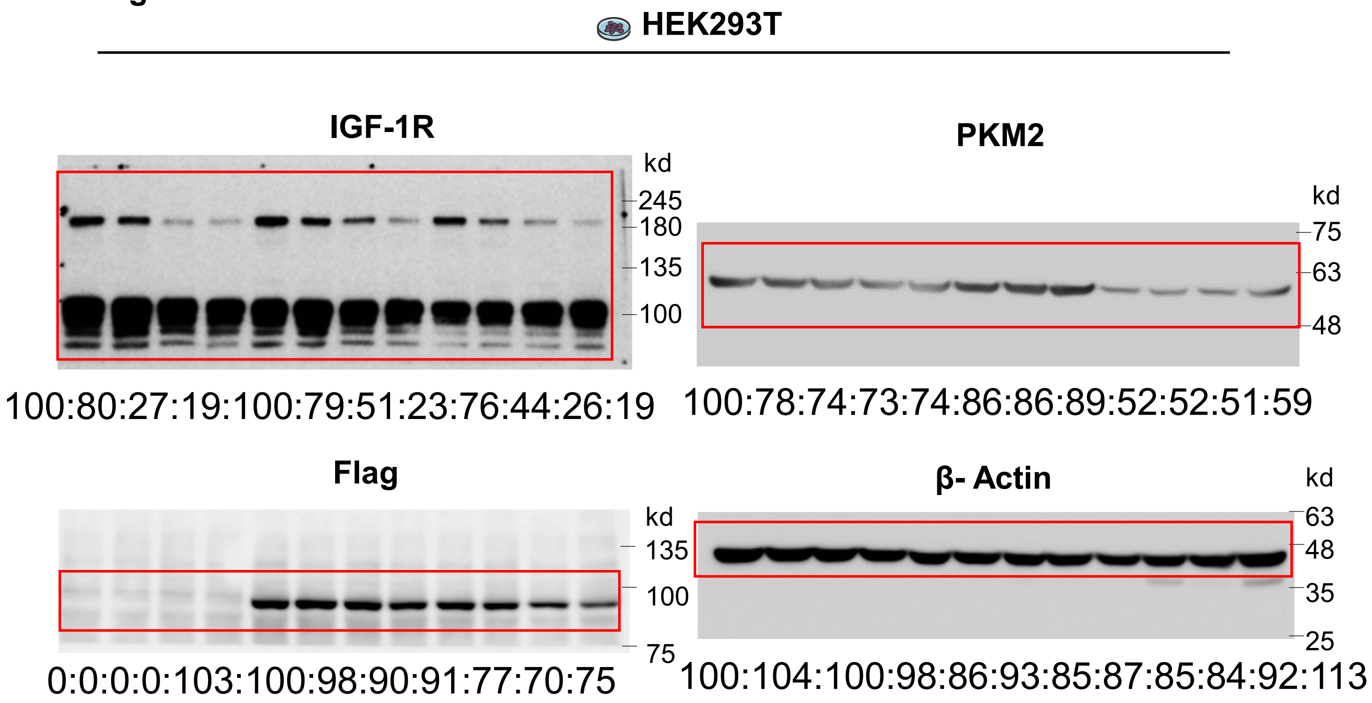

Fig. 4E

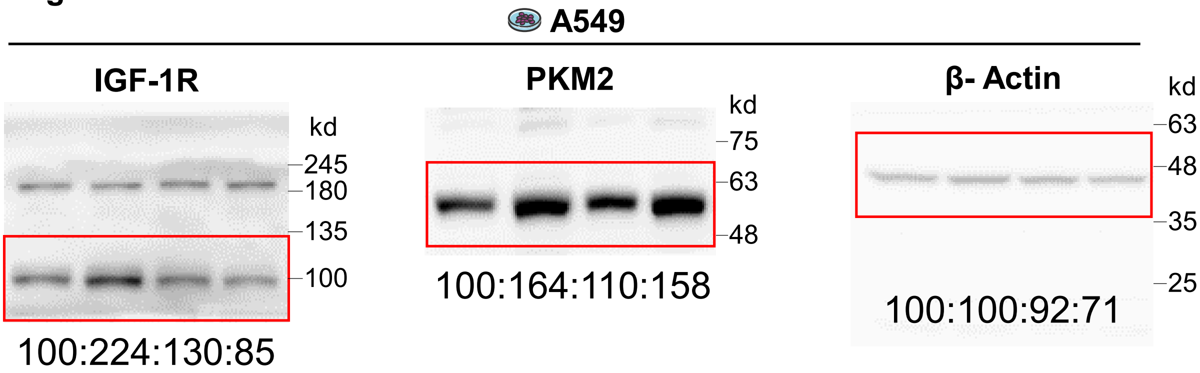

Fig. 4E

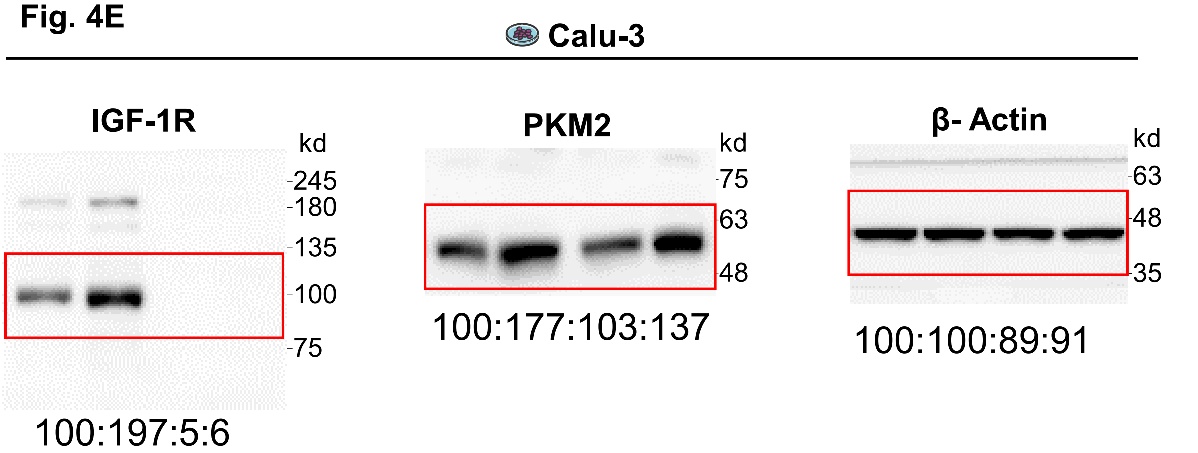

Full size immunoblots of cropped blots for Fig. 4D and 4E

Fig. S1B

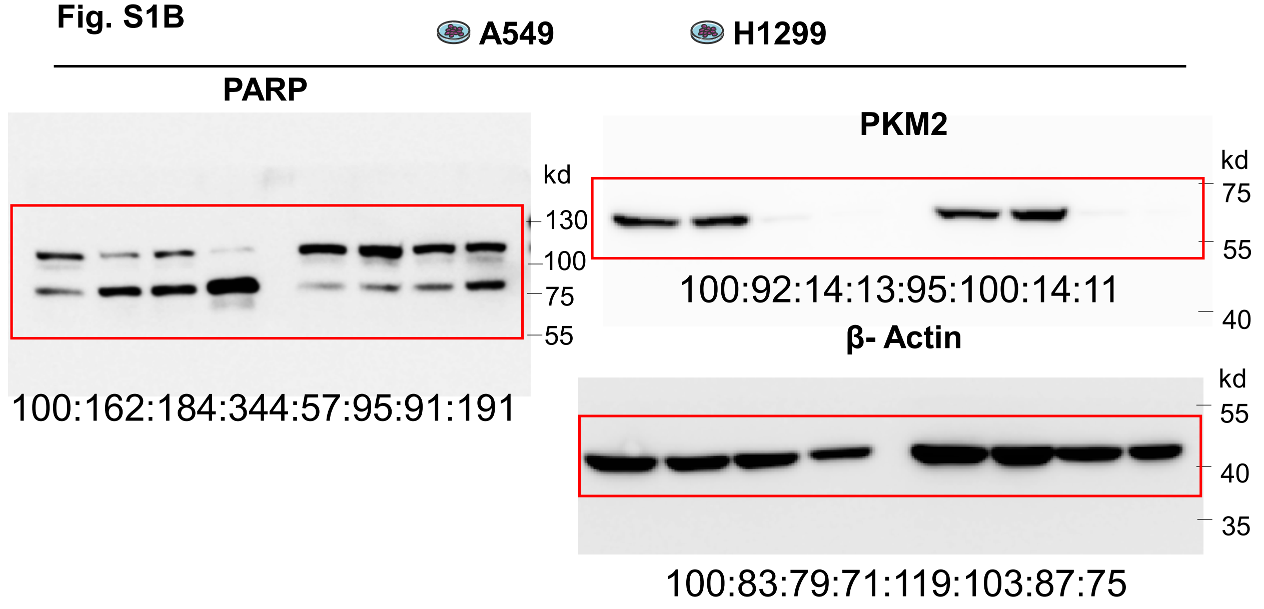

Fig. S2A

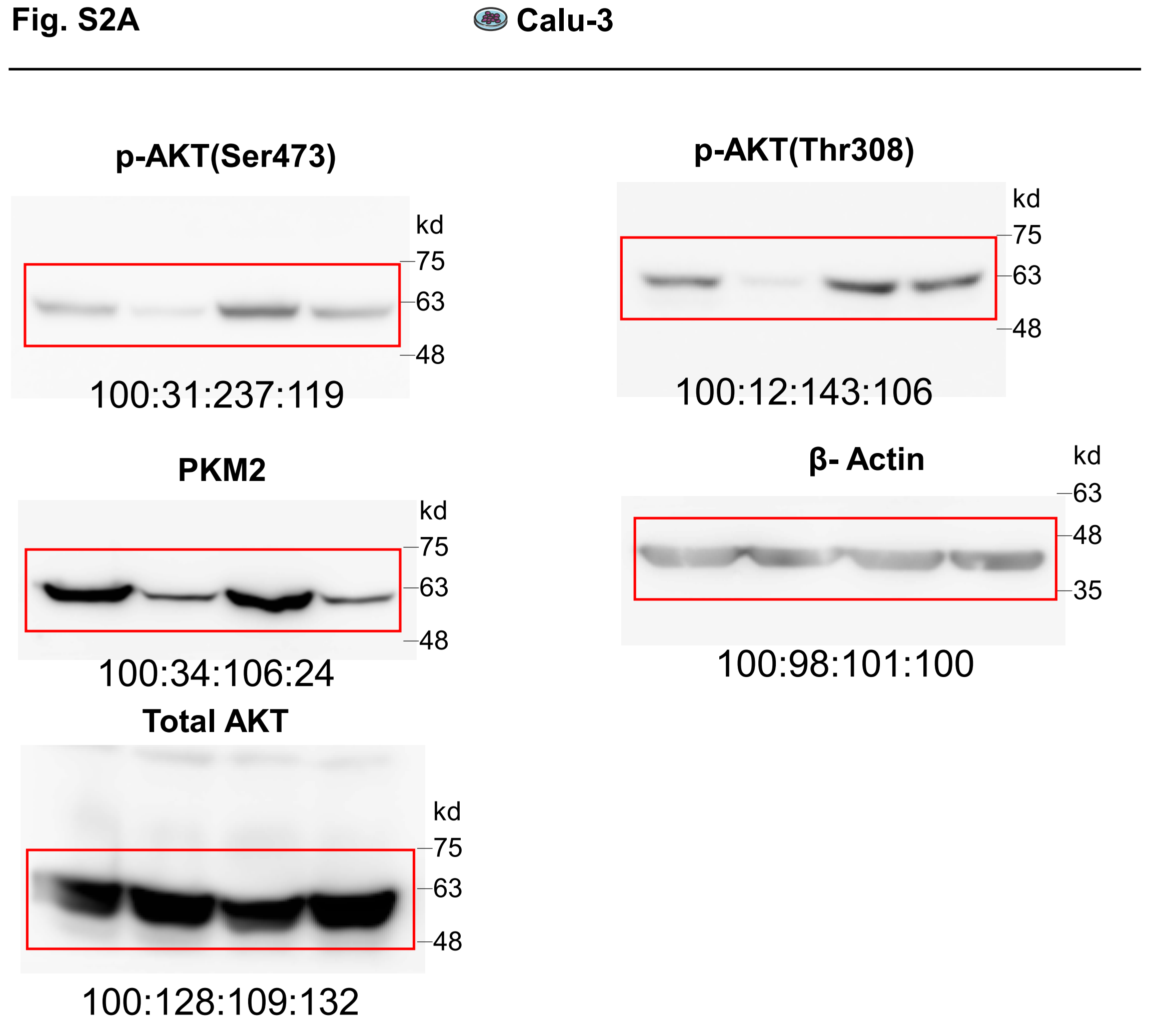

Full size immunoblots of cropped blots for Fig. S1B and S2A

Fig. S2B H1299

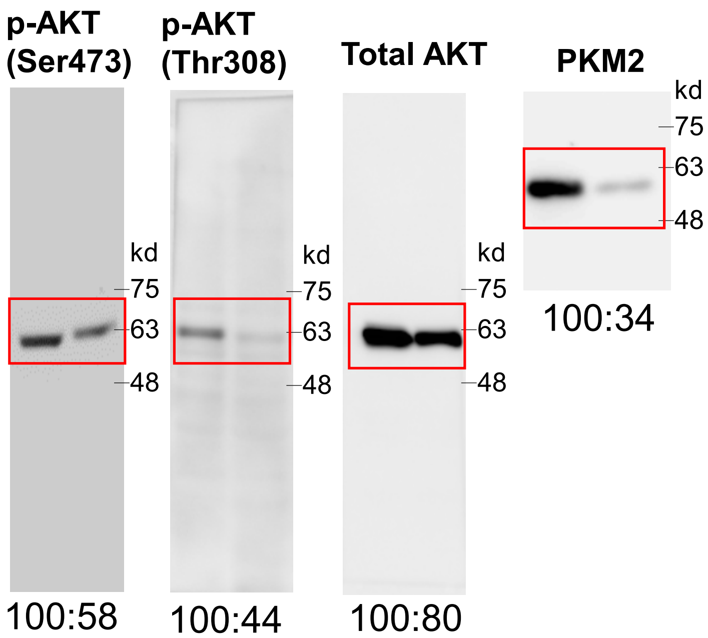

Fig. S2B H23

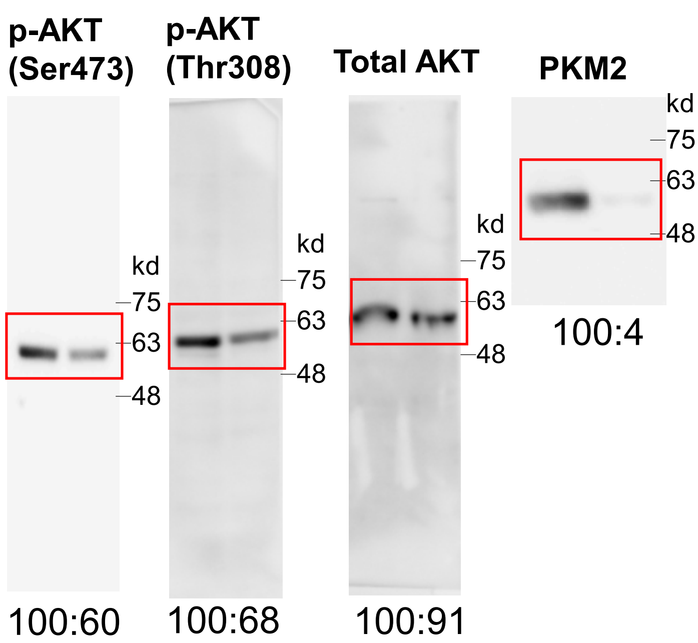

Fig. S2D A549 H1299

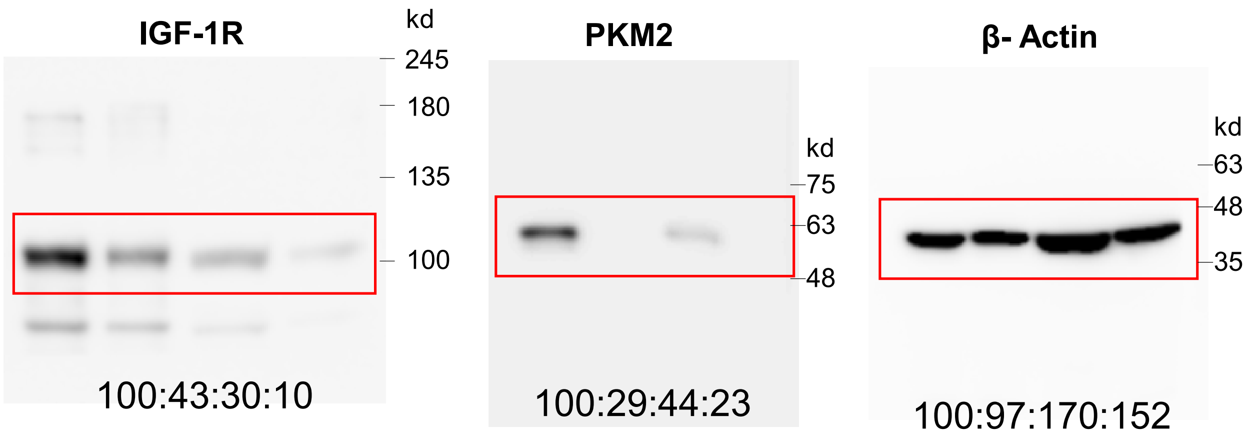

Fig. S2D H23

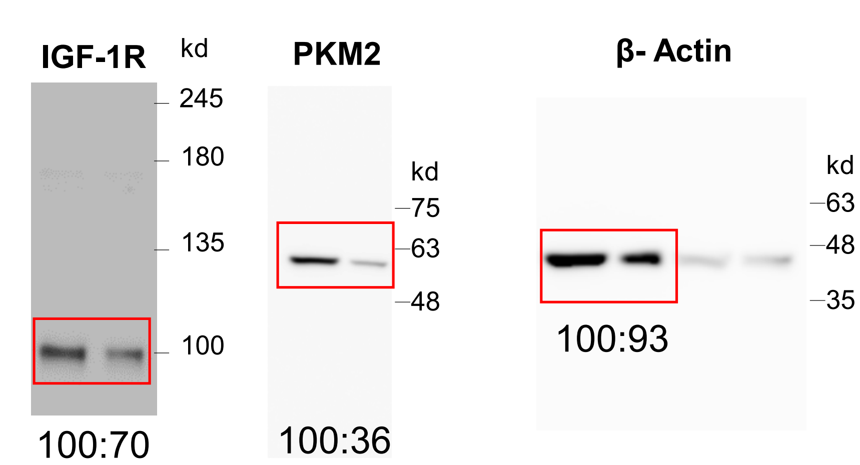

Full size immunoblots of cropped blots for Fig. S2B and S2D

Fig. S2G

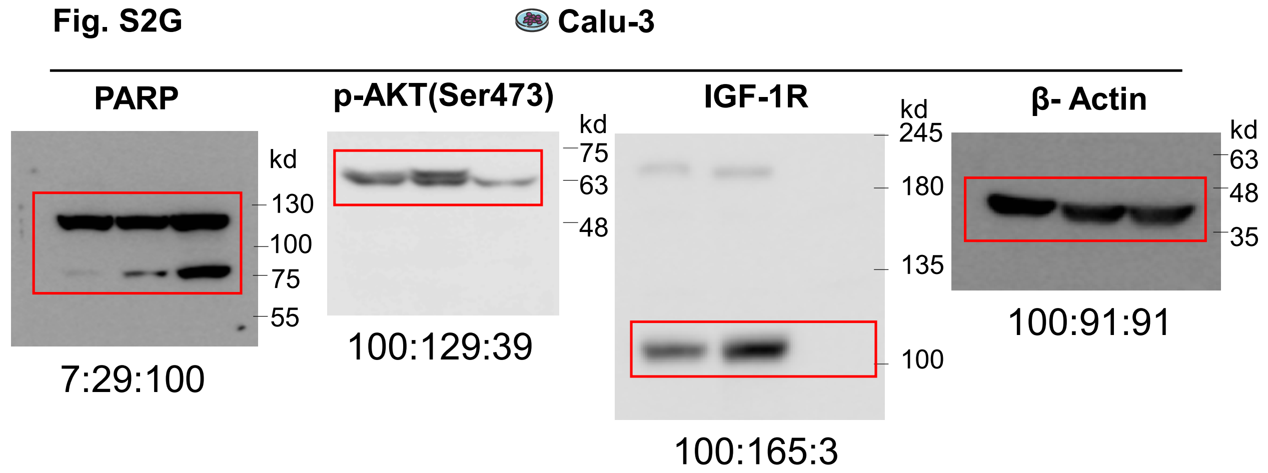

Fig. S2I

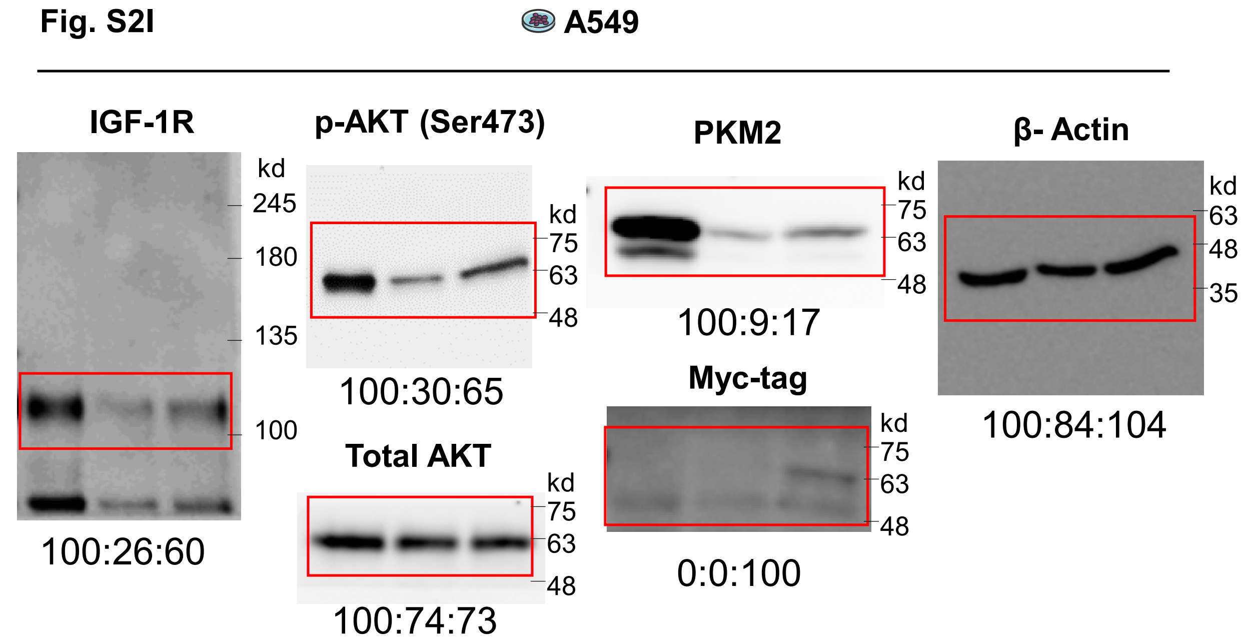

Fig. S2J

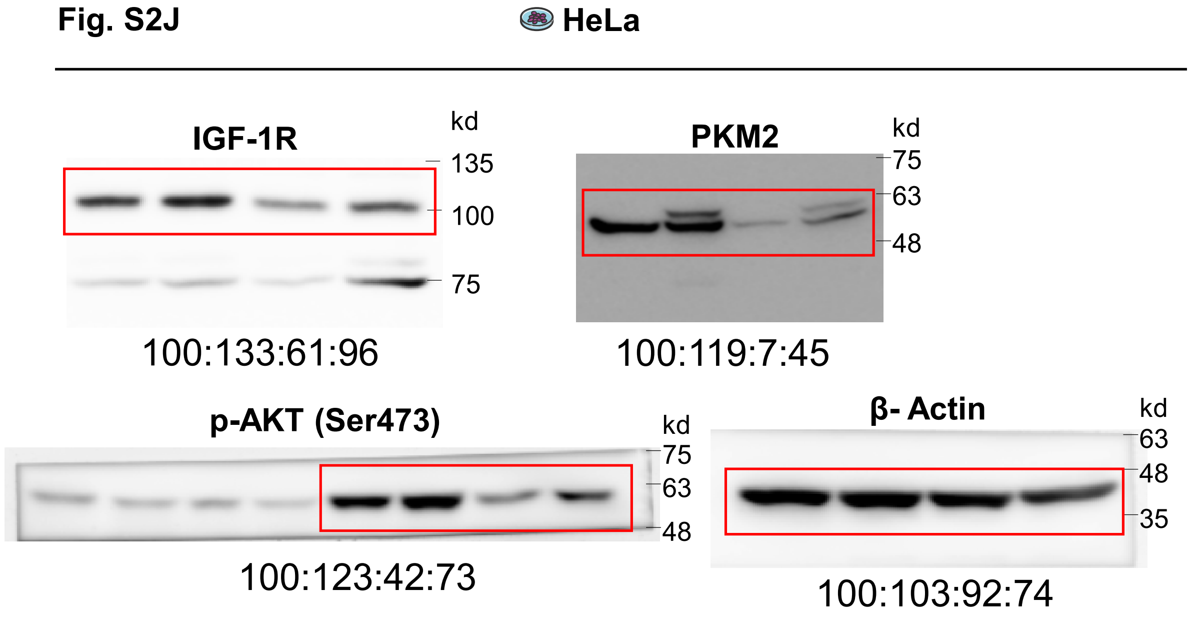

Full size immunoblots of cropped blots for Fig. S2G, S2I and S2J.

Fig. S2K **A549**

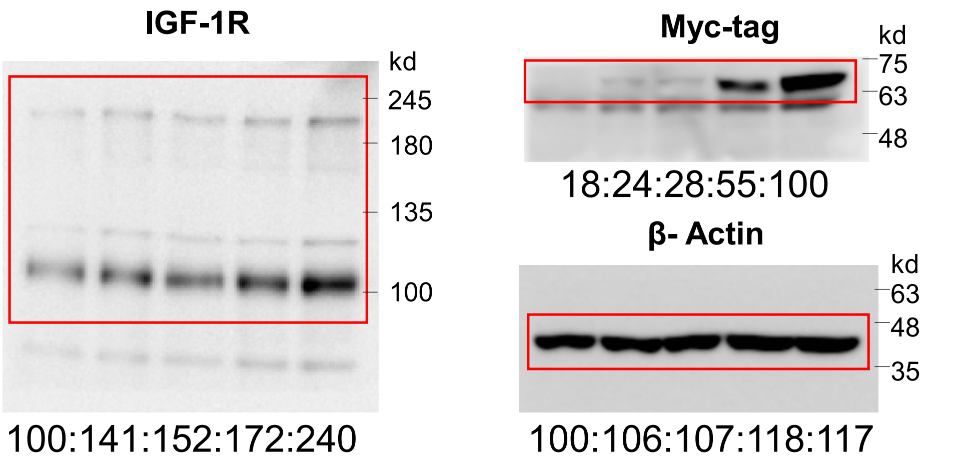

Fig. S2L **Calu-3**

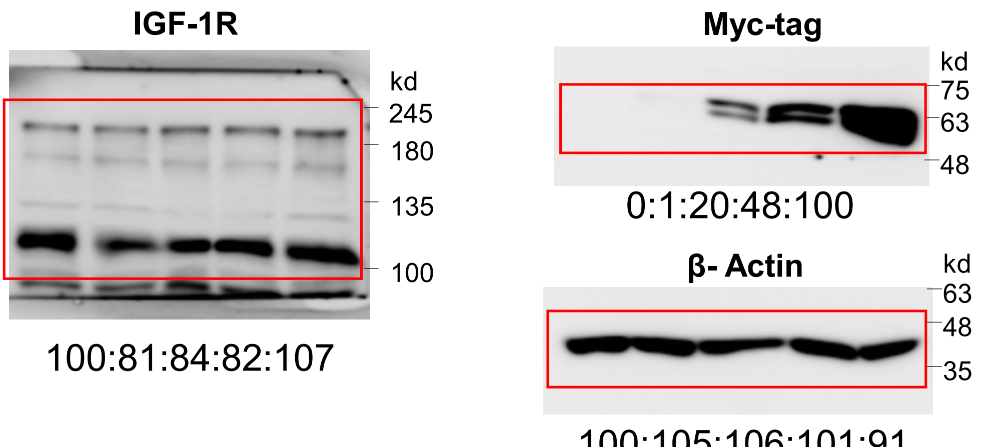

Fig. S2M **A549**

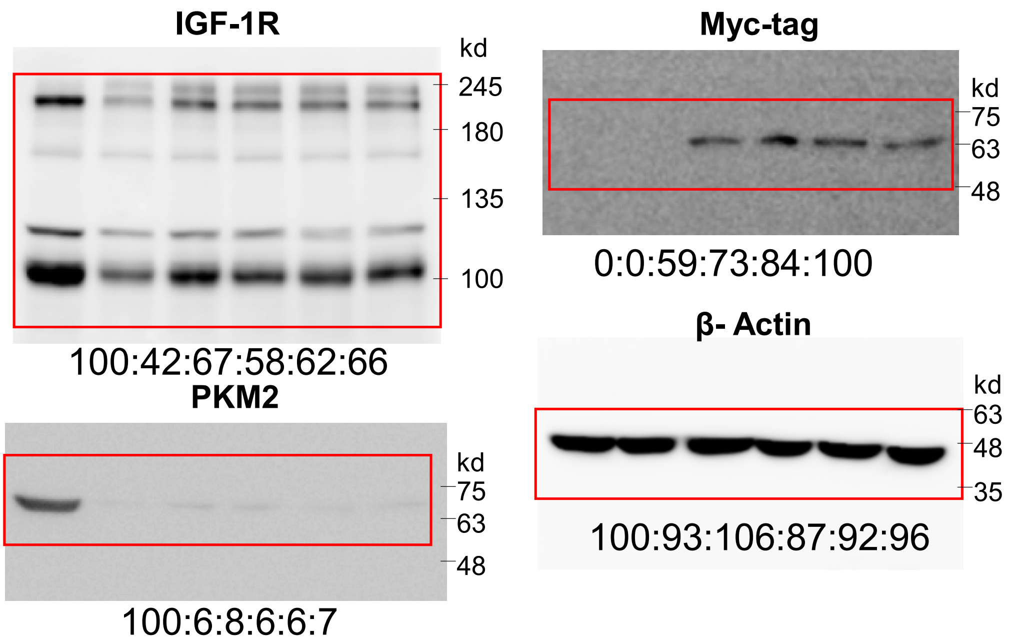

Full size immunoblots of cropped blots for Fig. S2K, S2L and S2M.

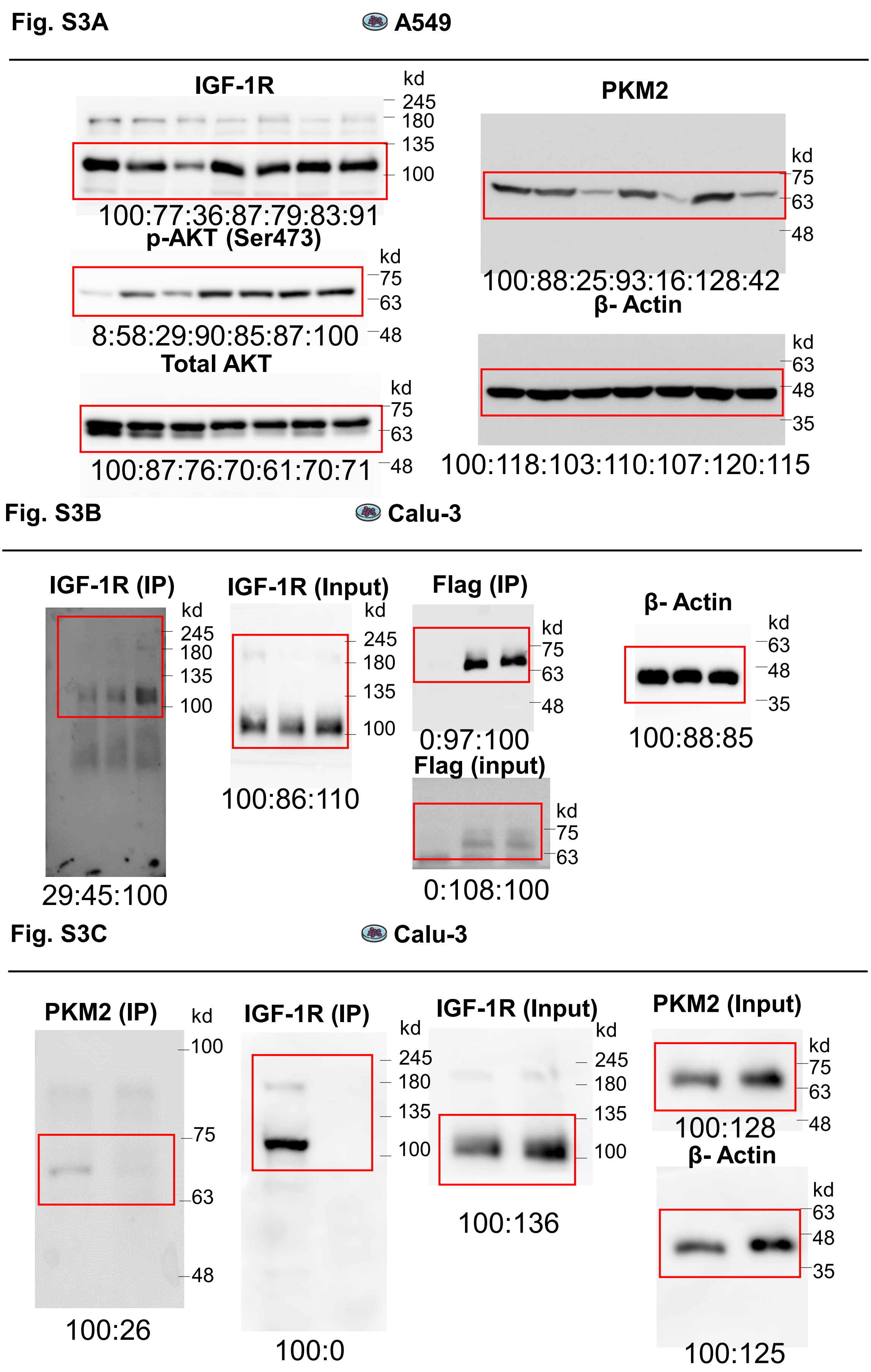

Full size immunoblots of cropped blots for Fig. S3A, S3B and S3C.

Fig. S3D A549

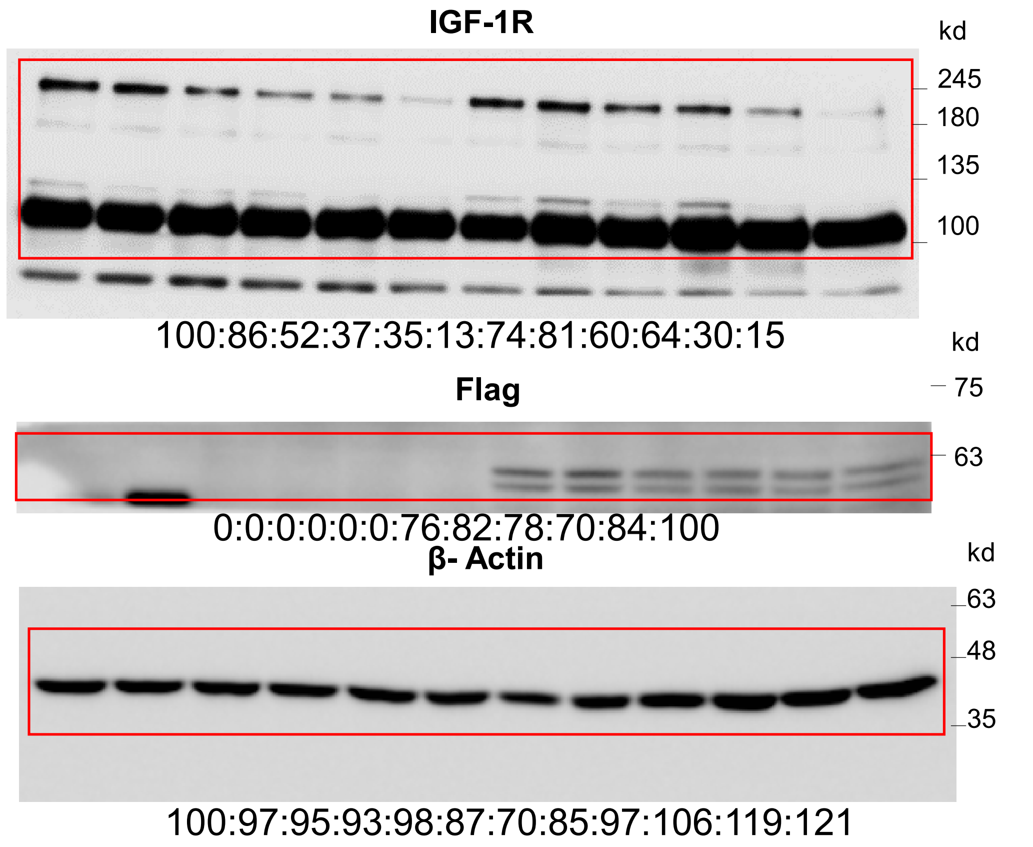

Fig. S4A Calu-3

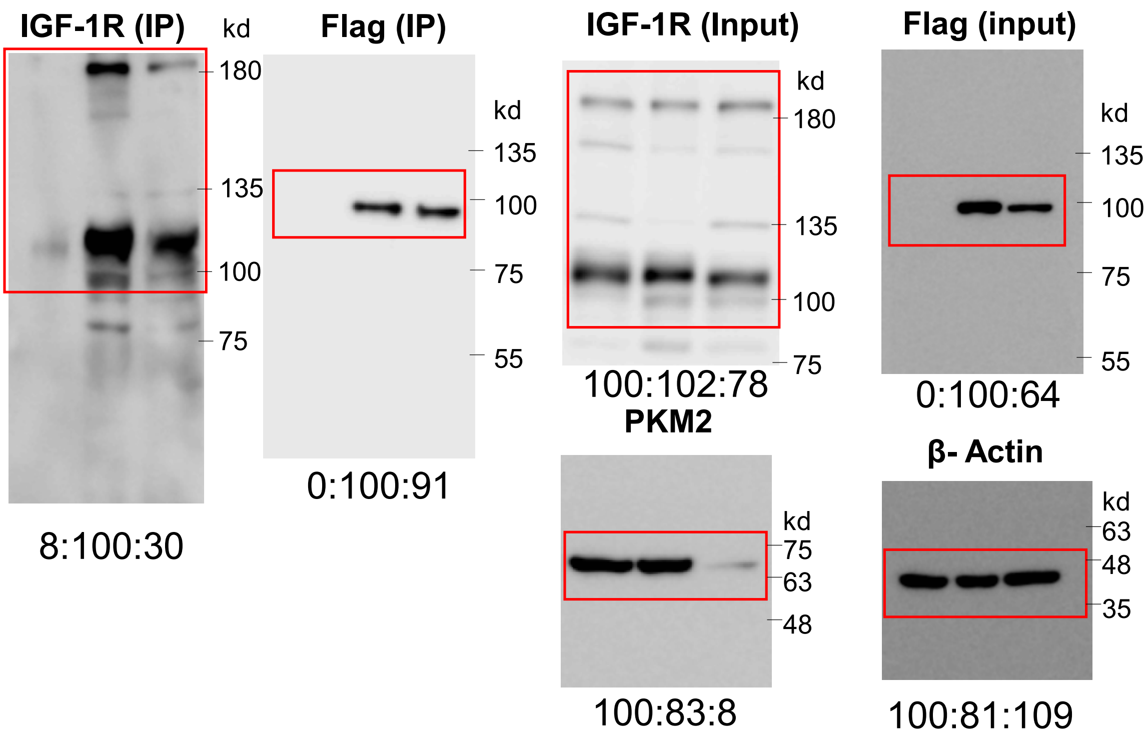

Full size immunoblots of cropped blots for Fig. S3D and S4A.

Fig. S4B A549

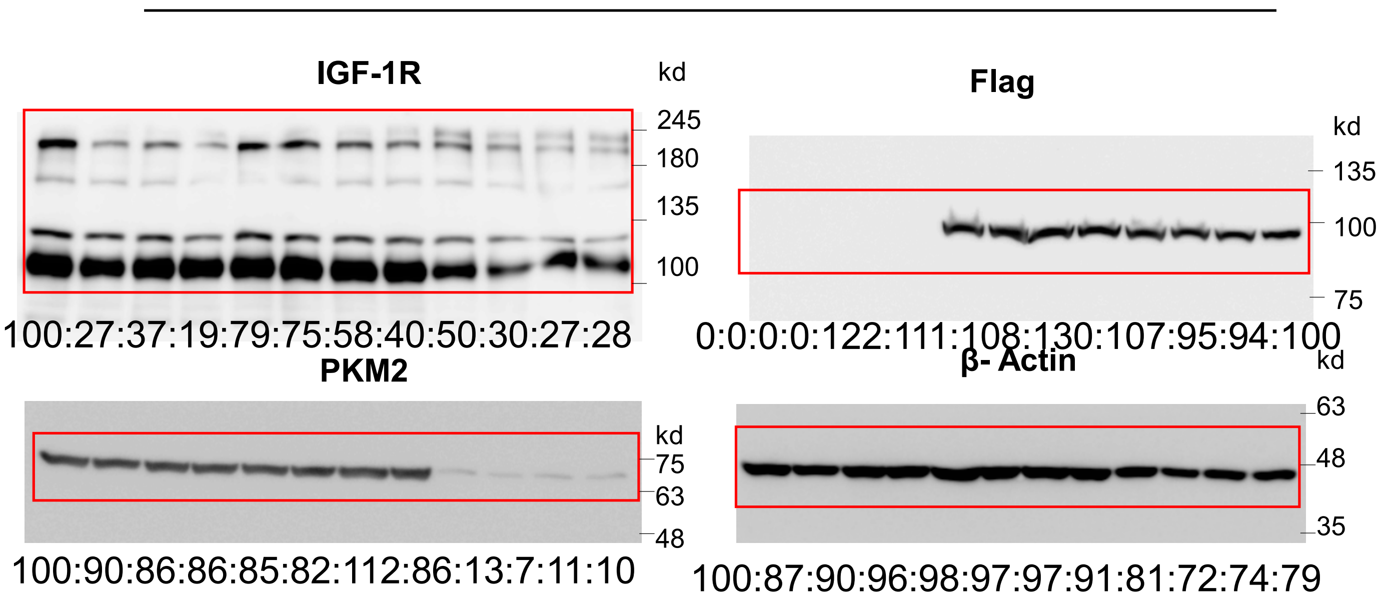

Fig. S4C H23

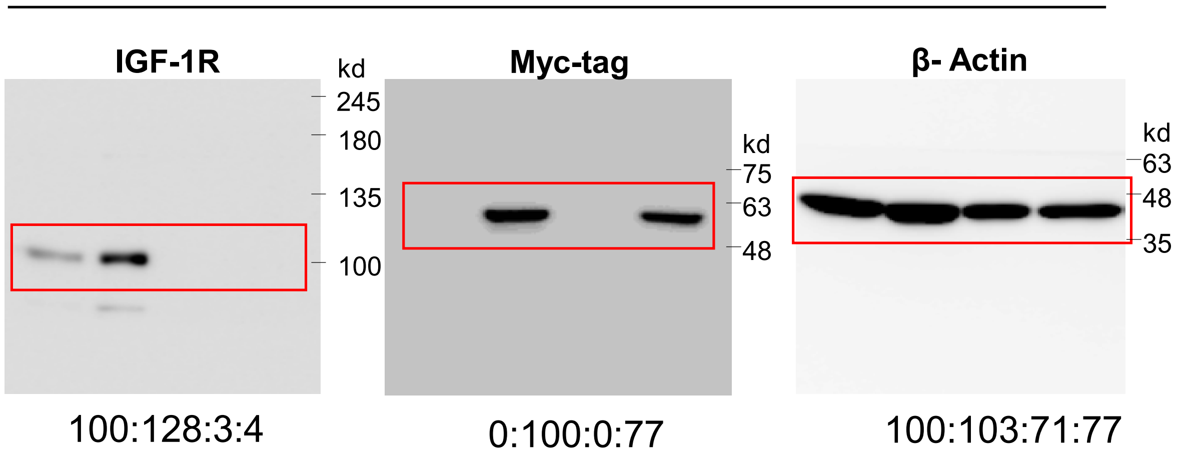

Fig. S4D H1299

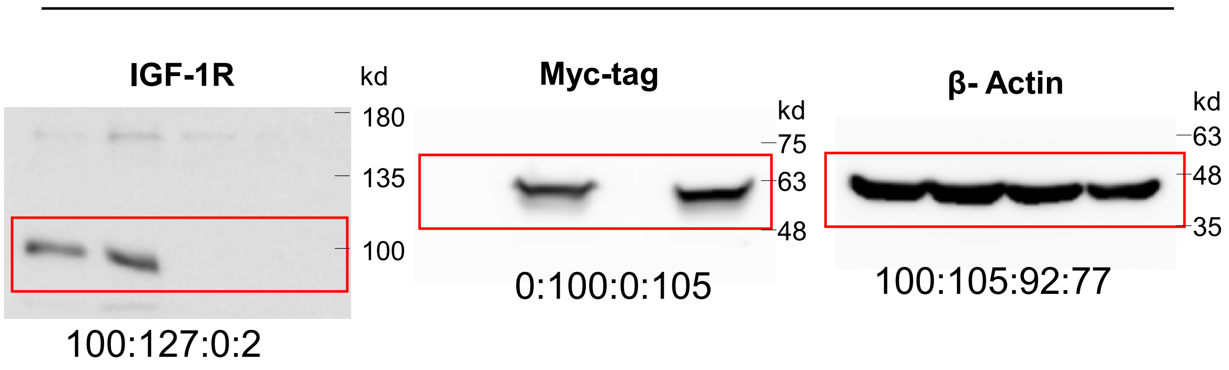

Full size immunoblots of cropped blots for Fig. S4B, S4C and S4D.
