# Supplementary material for: PKM2 Regulates HSP90-Mediated Stability of the IGF-1R Precursor Protein and Promotes Cancer Cell Survival during Hypoxia
Source: Cancers (Basel). 2021 Jul 30;13(15):3850. doi: 10.3390/cancers13153850 (PMC8345735; doi:10.3390/cancers13153850)
Supplement: Supplementary file 1 [file cancers-13-03850-s001.zip › Supplementary figures and tables_Koo et al_Final_2021-07-29.pdf]

Supplementary Materials

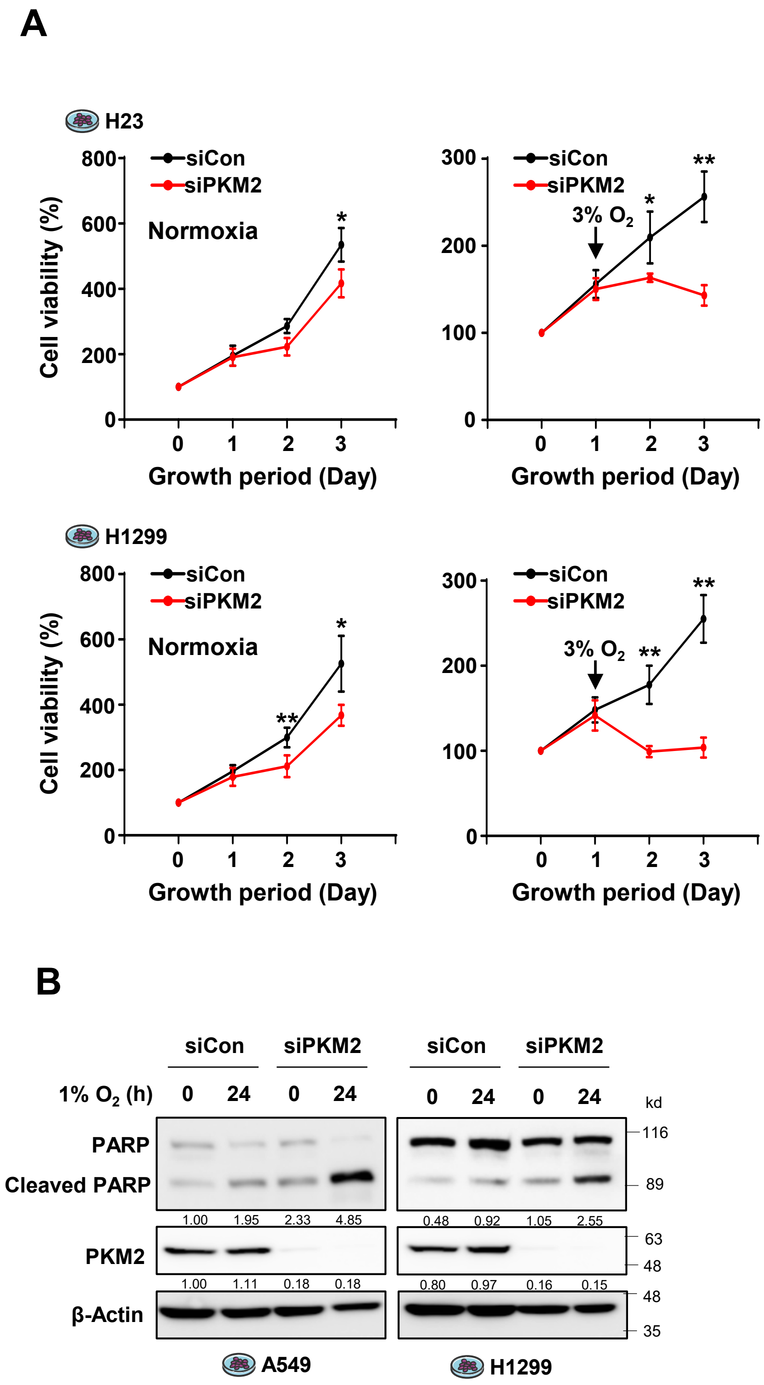

Figure S1. PKM2 deficiency reduces cell viability and induces apoptosis particularly in hypoxic condition. (**A**) Cell viability of H23 and H1299 cells transfected with siCon or siPKM2. Each cell was incubated in normoxia or 3% O_2 hypoxia for the indicated time. Cell numbers were determined by automated cell counter after staining with Trypan blue (n = 3). Statistical significance was measured using one-way ANOVA with the Tukey post hoc test. *P < 0.05, **P < 0.01. (**B**) The levels of PARP cleavage in A549 and H1299 cells transfected with siCon or siPKM2 under normoxic or hypoxic (1% O_2) conditions for 24h. β-Actin was used as a loading control.

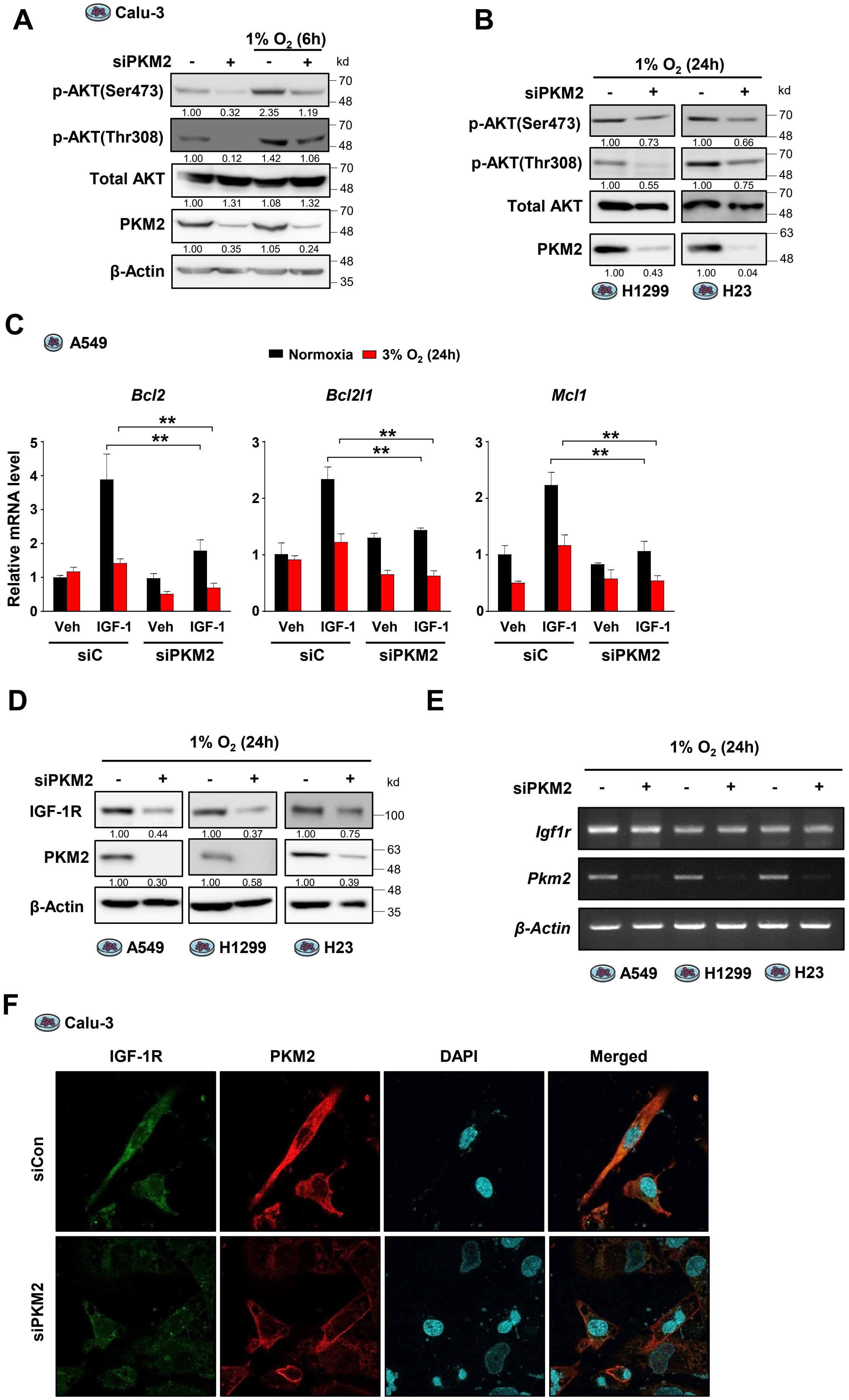
A
Calu-3
1% O2 (6h)
siPKM2 - + - +
kd
p-AKT(Ser473)
1.00 0.32 2.35 1.19
p-AKT(Thr308)
1.00 0.12 1.42 1.06
Total AKT
1.00 1.31 1.08 1.32
PKM2
1.00 0.35 1.05 0.24
β-Actin
70 48 70 48 70 48 70 48 48 35
B
1% O2 (24h)
siPKM2 - + - +
kd
p-AKT(Ser473)
1.00 0.73 1.00 0.66
p-AKT(Thr308)
1.00 0.55 1.00 0.75
Total AKT
PKM2
1.00 0.43 1.00 0.04
H1299 H23
70 48 70 48 70 48 63 48
C
A549
Normoxia 3% O2 (24h)
Bcl2
Bcl2l1
Mcl1
Relative mRNA level
**
Veh IGF-1 Veh IGF-1
siC siPKM2
D
1% O2 (24h)
siPKM2 - + - + - +
kd
IGF-1R
1.00 0.44 1.00 0.37 1.00 0.75
PKM2
1.00 0.30 1.00 0.58 1.00 0.39
β-Actin
100 63 48 48 35
A549 H1299 H23
E
1% O2 (24h)
siPKM2 - + - + - +
Igf1r
Pkm2
β-Actin
A549 H1299 H23
F
Calu-3
IGF-1R PKM2 DAPI Merged
siCon
siPKM2

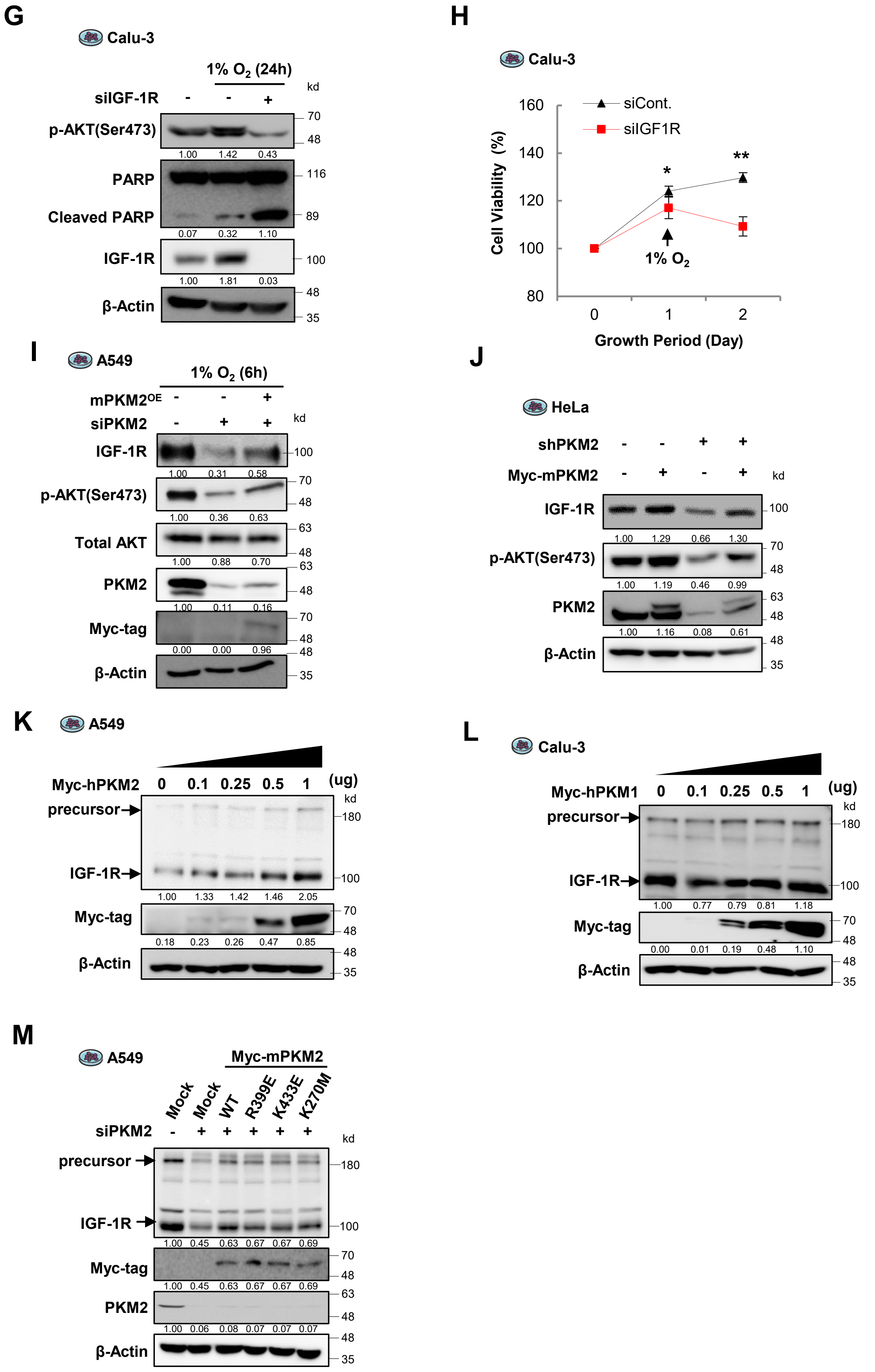

G
Calu-3
1% O2 (24h)
siIGF-1R - - +
kd
p-AKT(Ser473)
70
48
1.00 1.42 0.43
PARP
116
Cleaved PARP
89
0.07 0.32 1.10
IGF-1R
100
1.00 1.81 0.03
β-Actin
48
35
H
Calu-3
siCont.
siIGF1R
Cell Viability (%)
160
140
120
100
80
0 1 2
Growth Period (Day)
*
**
1% O2
I
A549
1% O2 (6h)
mPKM2OE - - +
siPKM2 - + +
kd
IGF-1R
100
1.00 0.31 0.58
p-AKT(Ser473)
70
48
1.00 0.36 0.63
Total AKT
63
48
1.00 0.88 0.70
PKM2
63
48
1.00 0.11 0.16
Myc-tag
70
48
0.00 0.00 0.96
β-Actin
48
35
J
HeLa
shPKM2 - - + +
Myc-mPKM2 - + - +
kd
IGF-1R
100
1.00 1.29 0.66 1.30
p-AKT(Ser473)
70
48
1.00 1.19 0.46 0.99
PKM2
63
48
1.00 1.16 0.08 0.61
β-Actin
48
35
K
A549
Myc-hPKM2 0 0.1 0.25 0.5 1 (ug)
kd
precursor
180
IGF-1R
100
1.00 1.33 1.42 1.46 2.05
Myc-tag
70
48
0.18 0.23 0.26 0.47 0.85
β-Actin
48
35
L
Calu-3
Myc-hPKM1 0 0.1 0.25 0.5 1 (ug)
kd
precursor
180
IGF-1R
100
1.00 0.77 0.79 0.81 1.18
Myc-tag
70
48
0.00 0.01 0.19 0.48 1.10
β-Actin
48
35
M
A549
Myc-mPKM2
Mock Mock WT R399E K433E K270M
siPKM2 - + + + + +
kd
precursor
180
IGF-1R
100
1.00 0.45 0.63 0.67 0.67 0.69
Myc-tag
70
48
1.00 0.45 0.63 0.67 0.67 0.69
PKM2
63
48
1.00 0.06 0.08 0.07 0.07 0.07
β-Actin
48
35

Figure S2. PKM2 deficiency reduces IGF-1R signaling-mediated cell survival by suppressing its expression in hypoxic conditions. (**A**) Phosphorylated AKT levels in Calu-3 cells transfected with siCon or siPKM2 under normoxic or hypoxic (1% O_2) conditions. (**B**) Phosphorylated AKT levels in H1299 and H23 cells transfected with siCon or siPKM2 under hypoxic (1% O_2) condition for 24h. Total AKT was used as a loading control. (**C**) The levels of mRNA expression of anti-apoptotic genes in A549 cells transfected with control siRNA or PKM2 siRNA under normoxic or hypoxic conditions (3% O2) for 24 h, normalized to β-actin mRNA. Values of graph were presented as mean ± SD. Statistical significance was measured using one-way ANOVA with the Tukey post hoc test. *P < 0.05, **P < 0.01. (**D**) Abundance of IGF-1R protein in A549, H1299 and H23 cells transfected with siCon or siPKM2 under hypoxic (1% O2) condition for 24 h. (**E**) The levels of IGF-1R mRNA expression in A549, H1299 and H23 cells transfected with siCon or siPKM2 under hypoxic (1% O2) condition for 24 h. **(F)** Another field of confocal microscopic images of endogenous IGF-1R and PKM2 detected by immunofluorescence in Calu-3 cells transfected with PKM2 siRNA under hypoxic condition (1% O2) for 6h. **(G)** Cleaved PARP, IGF-1R and phosphorylated AKT protein abundance in Calu-3 cells transfected with control siRNA or IGF-1R siRNA under normoxia or hypoxia (1% O_2) for 24 h. β-Actin was used as a loading control. **(H)** Cell viability of Calu-3 cells transfected with IGF-1R siRNA under normoxia and hypoxia (1% O_2) for the indicated periods. Cell numbers were measured by automated cell counting after staining with Trypan blue (n = 3). Values are presented as mean ± SD. Statistical significance was measured using the one-way ANOVA with the Tukey post-test. *P < 0.05 and **P < 0.01. **(I)** IGF-1R and p-AKT(S473) protein abundance in A549 cells transiently transfected with siPKM2 and/or Myc-tagged mouse PKM2 gene (Myc-mPKM2). **(J)** The effect of PKM2 restoration on IGF-1R protein expression in PKM2-depleted HeLa cells. IGF-1R and p-AKT(S473) protein abundance were determined in HeLa cells stably transfected with shPKM2 and transiently transfected with Myc-tagged mouse PKM2 gene (Myc-mPKM2) or mock vector. **(K)** Protein level of IGF-1R in A549 cells transiently transfected with different doses of Myc-tagged human PKM2 gene (Myc-hPKM2). **(L)** The effect of PKM1 overexpression on IGF-1R precursor or mature protein expressions. IGF-1R protein levels were determined in Calu-3 cells transiently transfected with different doses of Myc-tagged human PKM1 gene (Myc-hPKM1). **(M)** IGF-1R protein abundance in A549 cells transiently transfected with siPKM2 and/or Myc-tagged mutant mPKM2 genes. β-Actin was used as a loading control in **(A)**, **(D)**, **(E)**, **(G)** and **(I-M)**.

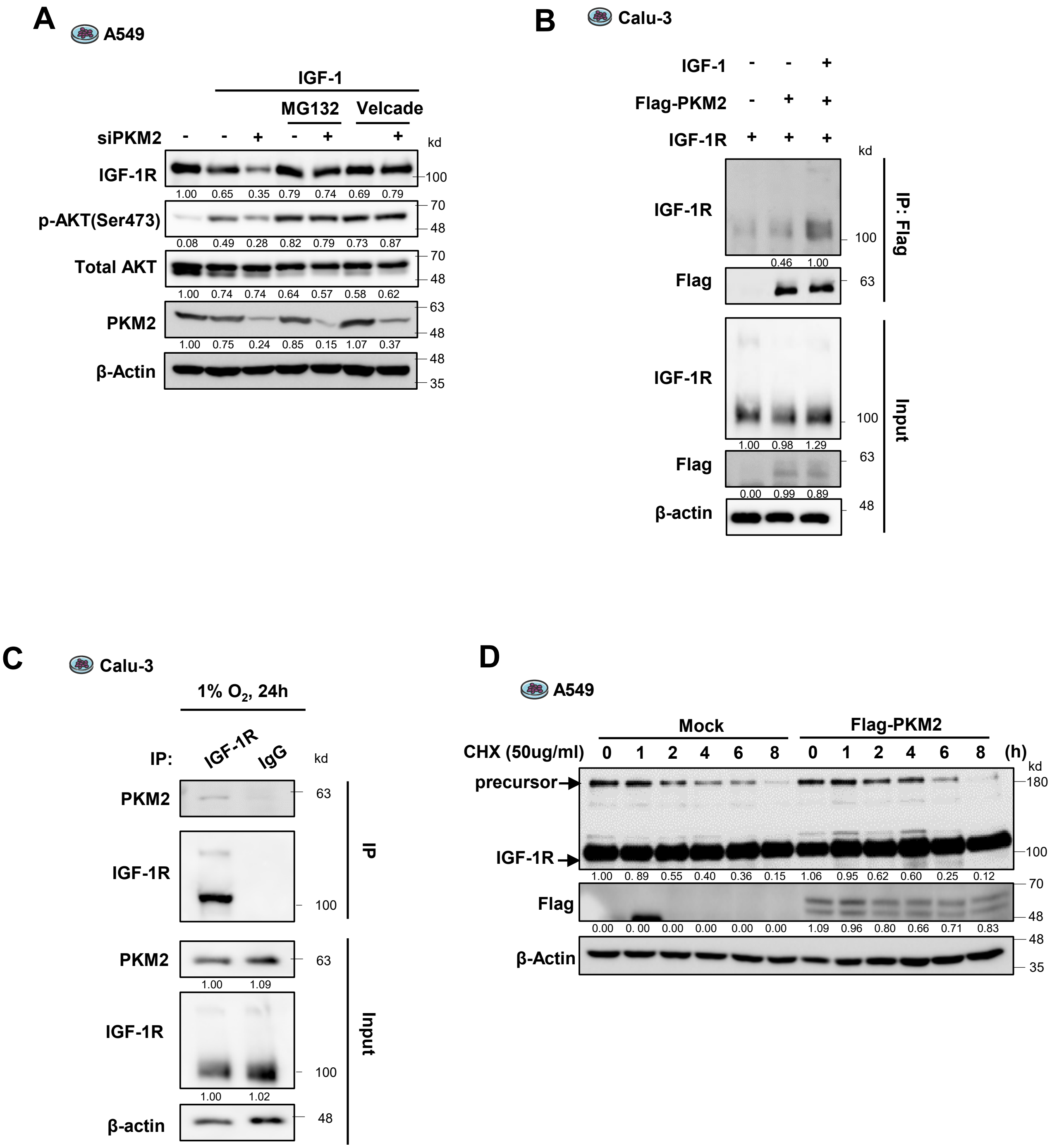
A
A549
IGF-1
MG132
Velcade
siPKM2
kd
IGF-1R
p-AKT(Ser473)
Total AKT
PKM2
β-Actin
1.00 0.65 0.35 0.79 0.74 0.69 0.79
0.08 0.49 0.28 0.82 0.79 0.73 0.87
1.00 0.74 0.74 0.64 0.57 0.58 0.62
1.00 0.75 0.24 0.85 0.15 1.07 0.37
100
70
48
63
35
B
Calu-3
IGF-1
Flag-PKM2
IGF-1R
Flag
β-actin
IP: Flag
Input
0.46 1.00
1.00 0.98 1.29
0.00 0.99 0.89
C
Calu-3
1% O2, 24h
IP:
IGF-1R
IgG
PKM2
β-actin
IP
Input
1.00 1.09
1.00 1.02
D
A549
Mock
Flag-PKM2
CHX (50ug/ml)
0 1 2 4 6 8 0 1 2 4 6 8 (h)
precursor
IGF-1R
Flag
β-Actin
180
1.00 0.89 0.55 0.40 0.36 0.15 1.06 0.95 0.62 0.60 0.25 0.12
0.00 0.00 0.00 0.00 0.00 0.00 1.09 0.96 0.80 0.66 0.71 0.83

Figure S3. PKM2 physically binds to IGF-1R and regulates its stability via the ubiquitin-proteasome system. **(A)** IGF-1R and p-Ser473-AKT protein abundance in siPKM2-expressing A549 cells pre-treated with the proteasome inhibitors, MG132 (10 μM) or Velcade (10 μM). **(B)** Abundance of IGF-1R precursor and mature proteins in the anti-Flag immunoprecipitates from Calu-3 cells exogenously expressing IGF-1R and Flag-PKM2. **(C)** Endogeneous PKM2 protein abundance in anti-IGF-1R immunoprecipitates from Calu-3 cells under hypoxic condition (1% O_2) for 24h. Normal mouse IgG was used as the negative control. **(D)** Determination of precursor and mature IGF-1R protein stability in Flag-PKM2 expressing A549 cells following CHX (50 μg/mL) treatment for the indicated time. β-Actin was used as the loading control in **(A-D)**.

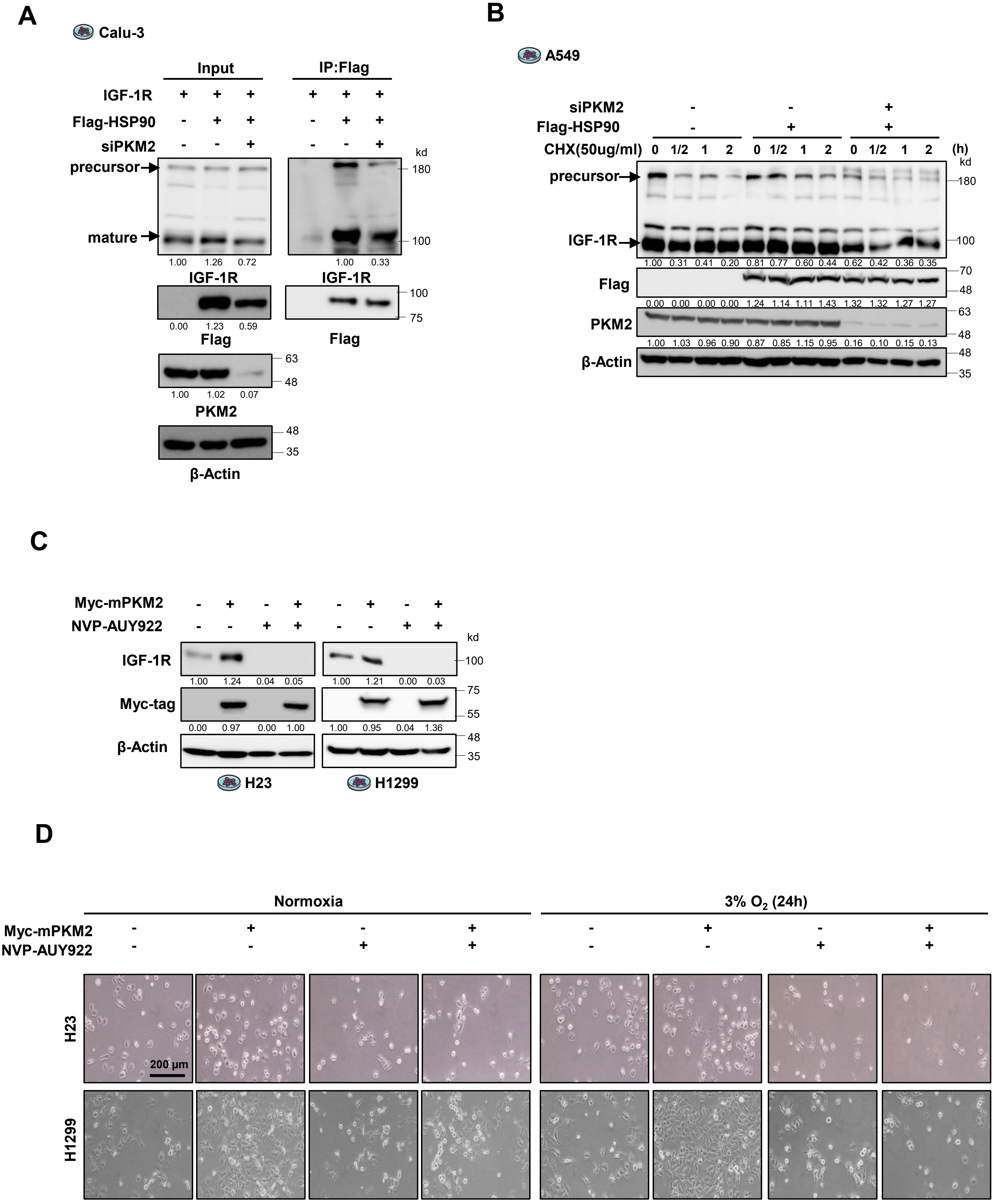
A
Calu-3
Input
IP:Flag
IGF-1R
Flag-HSP90
siPKM2
precursor
mature
kd
180
100
1.00 1.26 0.72
1.00 0.33
IGF-1R
0.00 1.23 0.59
Flag
63
48
1.00 1.02 0.07
PKM2
35
β-Actin
B
A549
siPKM2
Flag-HSP90
CHX(50ug/ml) 0 1/2 1 2 0 1/2 1 2 0 1/2 1 2 (h)
precursor
IGF-1R
1.00 0.31 0.41 0.20 0.81 0.77 0.60 0.44 0.62 0.42 0.36 0.35
Flag
0.00 0.00 0.00 0.00 1.24 1.14 1.11 1.43 1.32 1.32 1.27 1.27
PKM2
1.00 1.03 0.96 0.90 0.87 0.85 1.15 0.95 0.16 0.10 0.15 0.13
β-Actin
70
48
63
35
C
Myc-mPKM2
NVP-AUY922
IGF-1R
1.00 1.24 0.04 0.05
1.00 1.21 0.00 0.03
Myc-tag
0.00 0.97 0.00 1.00
1.00 0.95 0.04 1.36
β-Actin
75
55
H23
H1299
D
Normoxia
3% O2 (24h)
Myc-mPKM2
NVP-AUY922
H23
H1299
200 μm

Figure S4. PKM2 regulates the IGF-1R/HSP90 interaction contributing to cancer cell viability. **(A)** Abundance of IGF-1R precursor and mature proteins present in Flag-HSP90 immunoprecipitates from Calu-3 cells transfected with control siRNA or PKM2 siRNA. Each cell was also exogenously transfected with IGF-1R and Flag-HSP90 cDNAs, as indicated. **(B)** Precursor and mature IGF-1R protein stability assessed following treatment with CHX (50 ug/ml) for the indicated time in A549 cells expressing Flag-HSP90 and/or siPKM2. **(C)** IGF-1R protein levels were determined in H23 and H1299 cells transiently transfected with Myc-tagged mouse PKM2 gene (Myc-mPKM2) and subsequently treated with the HSP90 inhibitor NVP-AUY922 (500 nM) for 24 h. **(D)** The effect of NVP-AUY922 (500 nM) treatment on the cell viability was determined in Myc-mPKM2 expressing Calu-3 and A549 cells, cultured under normoxic conditions or 3% O2 for 24 h. The images were obtained using Olympus microscope IX71 with phase contrast. β-Actin was used as a loading control in **(A-C).**

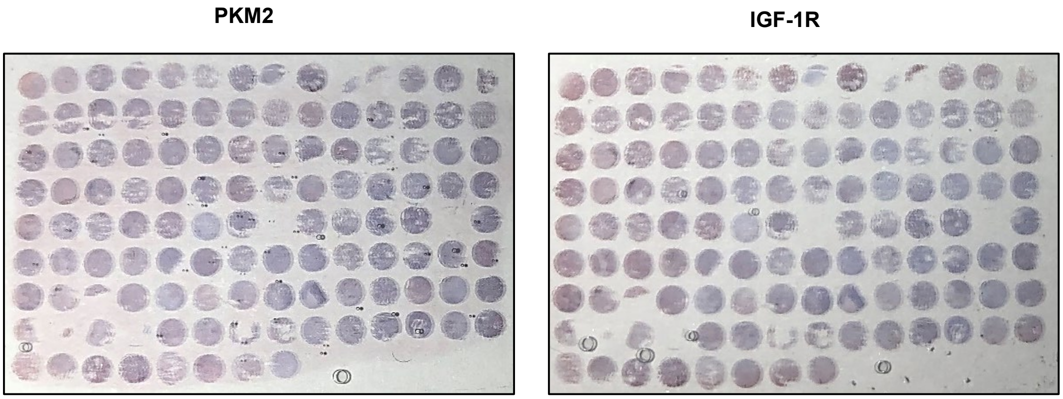

Figure S5. Whole membrane immunohistochemical images for PKM2 and IGF-1R expression in 112 human lung adenocarcinoma tissues.

Table S1. List of antibodies used in the study.

Target protein	Provider	Dilution factor
PKM2	Abnova (M01A)	WB(1:2000), ICC, IHC(1:200)
PKM2	Cell signaling (4053)	WB(1:2000)
IGF-1R	Abcam (ab182408)	ICC, IHC (1:200)
IGF-1R	Cell signaling (9750)	WB(1:1000)
Caspase-9	Cell signaling (9502)	WB(1:1000)
PARP	Cell signaling (9542)	WB(1:1000)
HIF-1a	BD bioscience (610958)	WB(1:1000)
ß-Actin	Santa Cruz (sc-47778)	WB(1:2000)
p-AKT(ser308)	Cell signaling (4056)	WB(1:500)
p-AKT(ser473)	Cell signaling (4060)	WB(1:2000)
AKT	Cell signaling (9272)	WB(1:1000)
Myc-tag	Cell signaling (2278)	WB(1:1000)
HA-tag	Cell signaling (3724)	WB(1:1000)
Flag-tag	Sigma (F3165)	WB(1:5000)
K48-ubiquitin	Cell signaling(8081)	WB(1:1000)

Table S2. List of primer sequence used in RT-PCR analysis.

Target gene	Sequence
Pkm2	Forward : 5'-CCGCCGCCTGGCGCCCATTA-3'<br>Reverse: 5'-CGGTCAGCACAATGACCACATC-3'
Igf1r	Forward : 5'- TGAGGATCAGCGAGAATGTG-3'<br>Reverse: 5'-CAGAGGCATACAGCACTCCA-3'
ß-Actin	Forward : 5'-CTGGAGAAGAGCTACGAGCTGC-3'<br>Reverse : 5'-CTAGAAGCATTTGCGGTGGACG-3'
Bcl2	Forward : 5'-GATGTGATGCCTCTGCGAAG-3'<br>Reverse : 5'-CATGCTGATGTCTCTGGAATCT-3'
Bcl2l1	Forward : 5'-GAGCTGGTGGTTGACTTTCTC-3'<br>Reverse : 5'-TCCATCTCCGATTCAGTCCCT-3'
Mcl1	Forward : 5'-AGAAAGCTGCATCGAACCAT-3'<br>Reverse : 5'-CCAGCTCCTACTCCAGCAAC-3'
