# Supplementary figures and images for: PKM2 Regulates HSP90-Mediated Stability of the IGF-1R Precursor Protein and Promotes Cancer Cell Survival during Hypoxia
Source: Cancers (Basel). 2021 Jul 30;13(15):3850. doi: 10.3390/cancers13153850 (PMC8345735; doi:10.3390/cancers13153850)

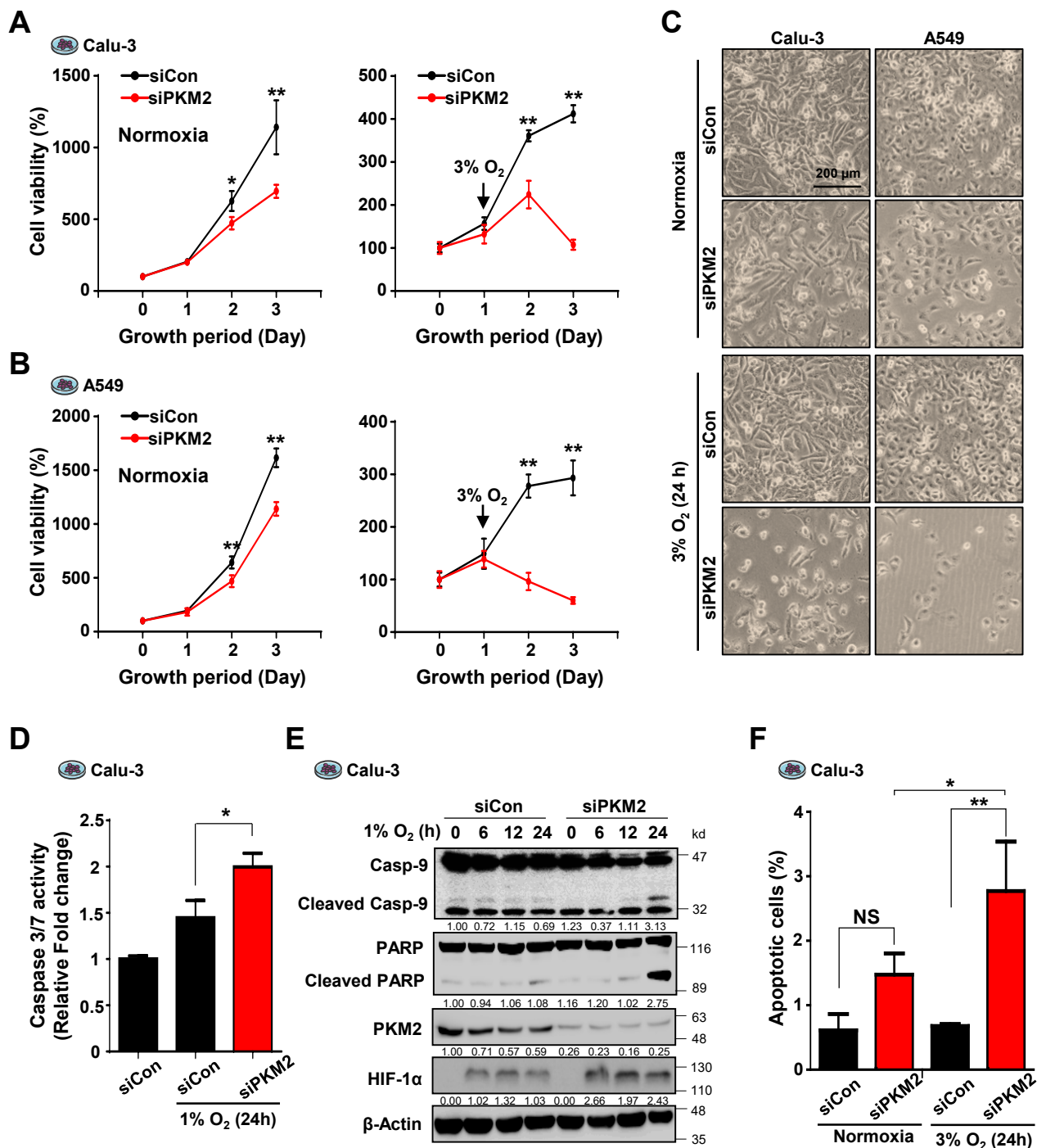

Figure 1. Koo *et al.*

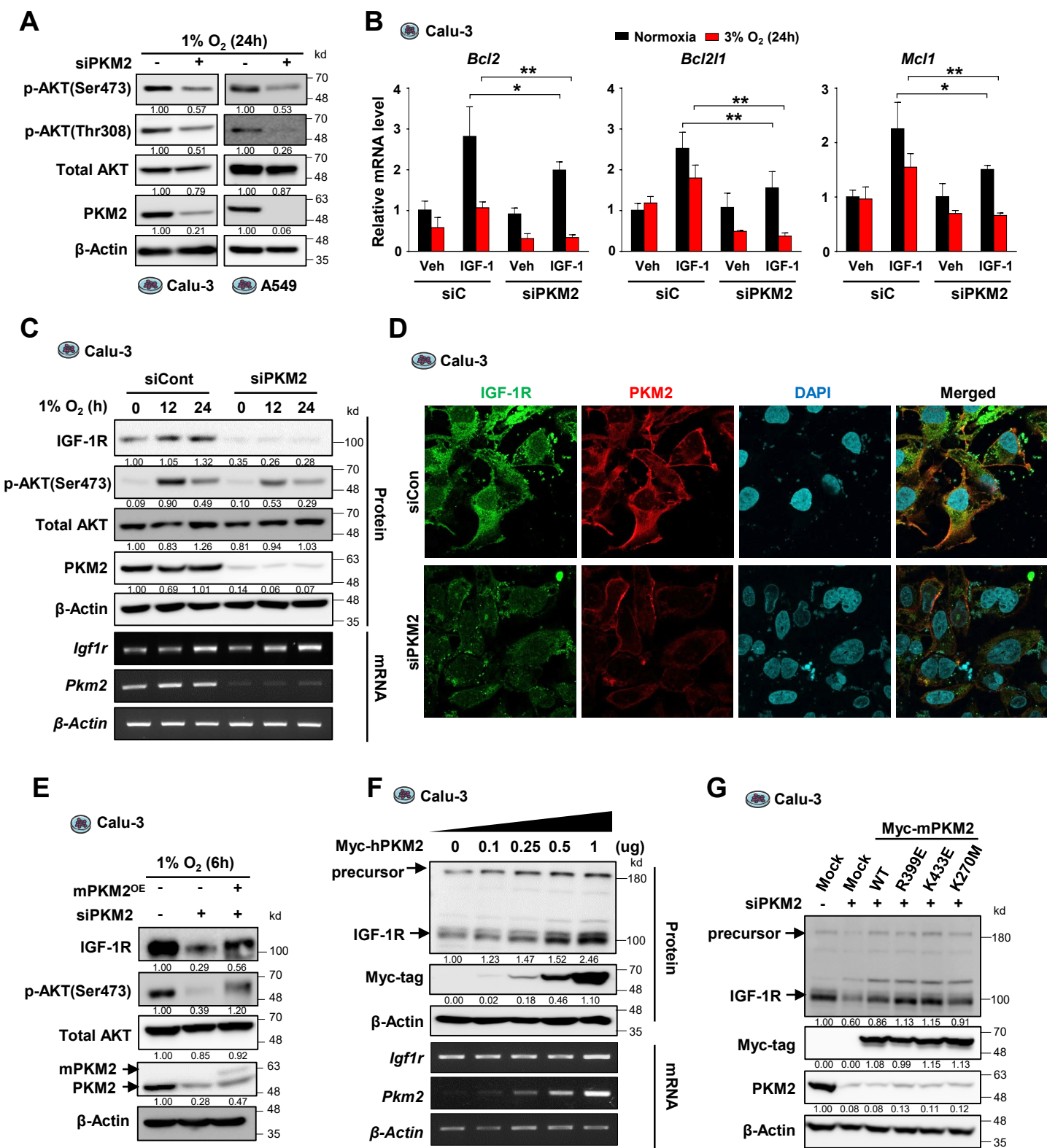

Figure 2. Koo et al.

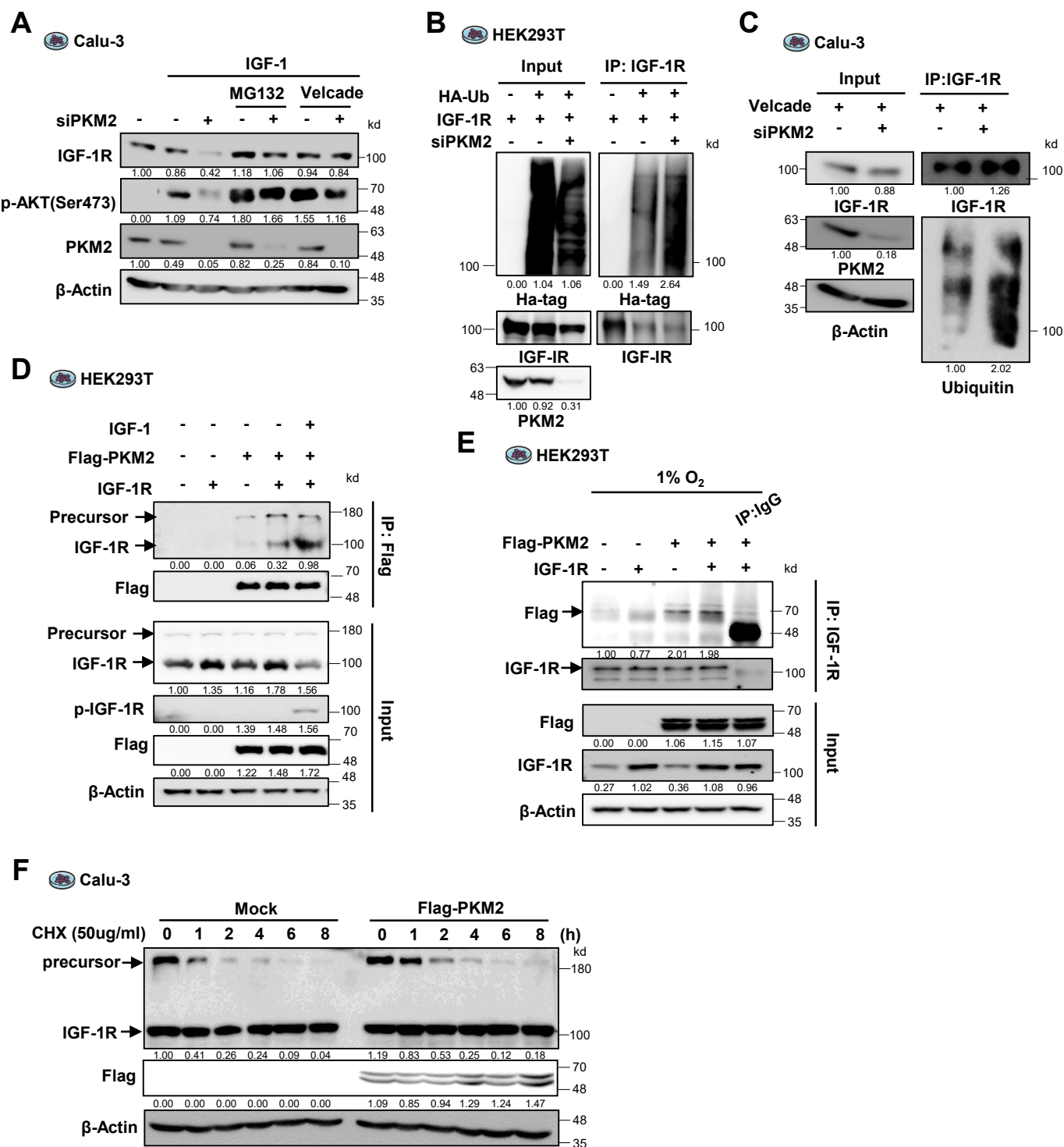

Figure 3. Koo *et al.*

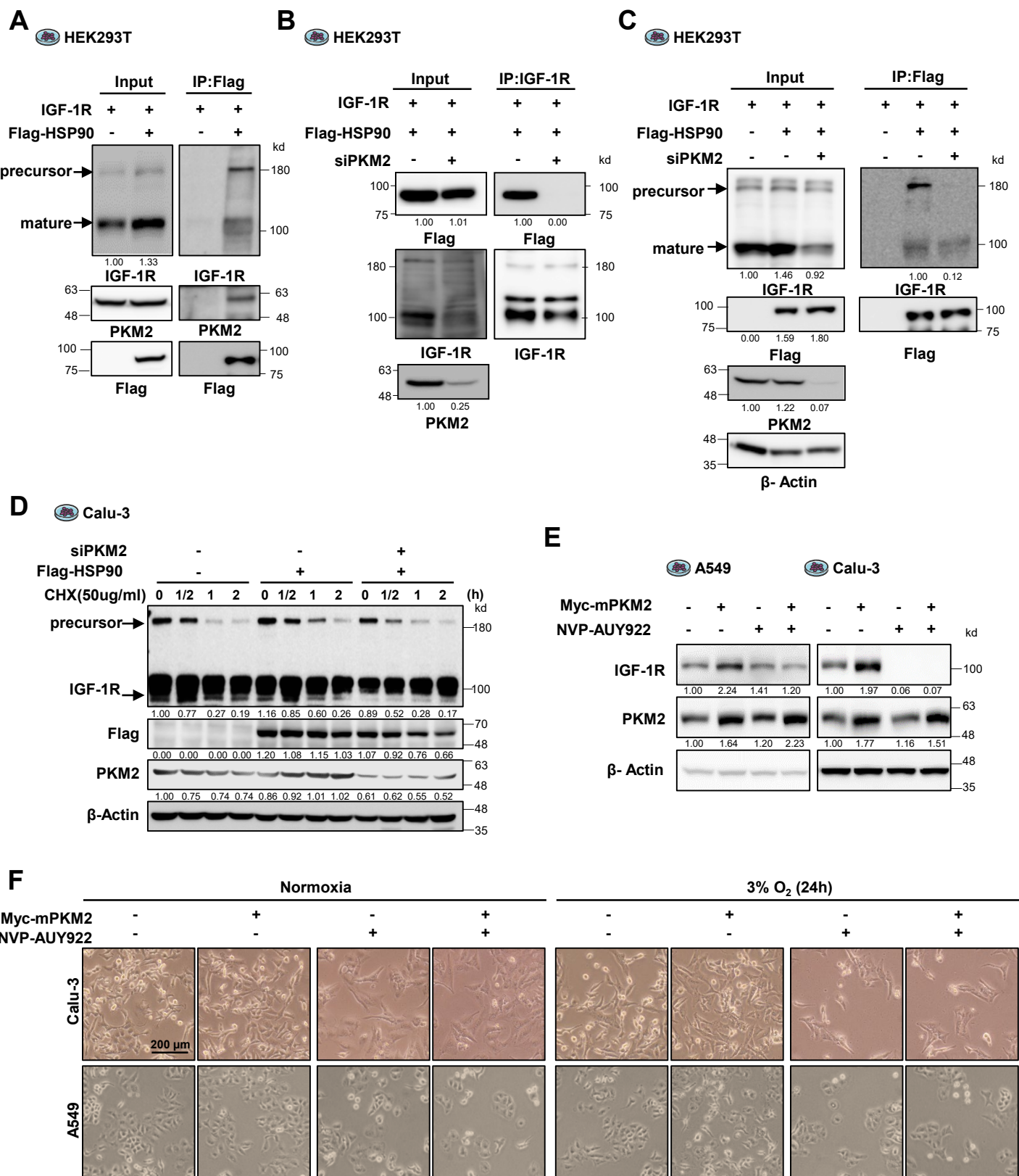

Figure 4. Koo *et al.*

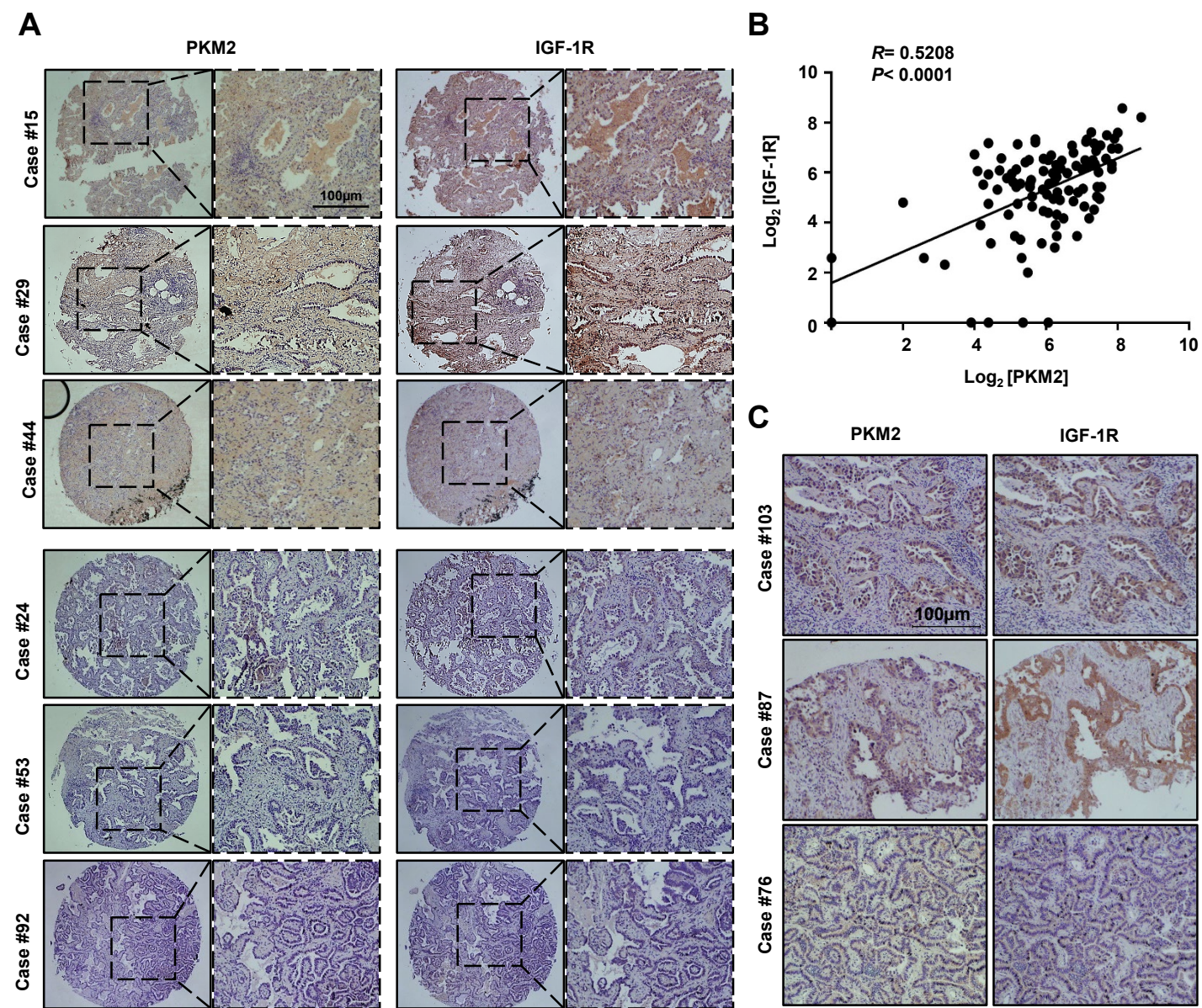

Figure 5. Koo *et al.*

Supplement: Supplementary file 1 [file cancers-13-03850-s001.zip › Main figures_PKM2_IGF-1R_Koo et al_Final_2021-07-27.pdf]
